# Modal Analysis with Asymptotic Strips Boundary Conditions of Skewed Helical Gratings on Dielectric Pipes as Cylindrical Metasurfaces for Multi-Beam Holographic Rod Antennas

**DOI:** 10.3390/s24248119

**Published:** 2024-12-19

**Authors:** Malcolm Ng Mou Kehn, Ting-Wei Lin, Wei-Chuan Chen

**Affiliations:** Institute of Communications Engineering, National Yang Ming Chiao Tung University, Hsinchu 30010, Taiwan; david2000891202.ee12@nycu.edu.tw (T.-W.L.); jo3fm06.ee12@nycu.edu.tw (W.-C.C.)

**Keywords:** asymptotic boundary conditions, cylindrical antennas, dielectric rod antennas, helical strip gratings, vector potential analysis, modal analysis, holographic antennas

## Abstract

A core dielectric cylindrical rod wrapped in a dielectric circular pipe whose outer surface is enclosed by a helical conducting strip grating that is skewed along the axial direction is herein analyzed using the asymptotic strip boundary conditions along with classical vector potential analysis. Targeted for use as a cylindrical holographic antenna, the resultant field solutions facilitate the aperture integration of the equivalent cylindrical surface currents to obtain the radiated far fields. As each rod section of a certain skew angle exhibits a distinct modal attribute; this topology allows for the distribution of the cylindrical surface impedance via the effective refractive index to be modulated, as in gradient-index (GRIN) materials. Beam steering can also be achieved by altering the skew angle via mechanical sliding motion while leaving the cylindrical structure itself unchanged, as opposed to impractically reconfiguring the geometrical and material parameters of the latter to attain each new beam direction. The results computed by the program code based on the proposed technique in terms of the modal dispersion and radiation patterns are compared with simulations by a software solver. Manufactured prototypes are measured, and experimentally acquired dispersion diagrams and radiation patterns are favorably compared with theoretical predictions.

## 1. Introduction

One of the earliest known uses of a dielectric rod can be traced back to [1], in which the transport of electromagnetic energy from an excitation feed to an output port by a supported propagating mode was reported. Besides applications such as waveguides, these cylindrical structures have also served widely as cylindrical antennas [2,3,4,5,6]. The main advantages of dielectric rods include their wide bandwidth, low polarization cross-coupling, and low cost [7,8]. The importance of these benefits is accentuated in the context of modern and future communications systems that are extended to millimeter-wave (mm wave) bands in response to demands for wider bandwidths and higher speeds [9].

Metasurfaces are artificially textured surfaces with two-dimensional (2D) lattices and composed of unit cells which encompass combinations of metallic and/or dielectric elements with periodicities that are much smaller than the operating wavelength [10]. Although most are typically planar, cylindrical forms of such surfaces wrapped around dielectric rods to create what are known as metasurface-loaded dielectric rod antennas have been investigated [11,12,13]. Cylindrical rods with periodically textured surfaces have also found use as electromagnetic bandgap (EBG) structures [14,15,16,17,18]. They can also be applied as cylindrical arrays [19,20,21] for base station or local area network (LAN) antennas. Besides waveguides and antennas, such structures have also been studied as mantle cloaks [22], which bend impingent waves around their curved surfaces, thereby rendering the regions within them effectively invisible at the operating frequency. Compared to the original forms, known as metamaterials [23], which are composed typically of three-dimensional lattices, the advantages of thin metasurfaces include ease and low cost of fabrication, as well as reduced weight and volume.

Aside from textures with the constitutional two-dimensional periodicity, the one-dimensional (1D) versions of flat metasurfaces comprising conducting strip gratings etched on dielectric surfaces have also attracted considerable attention, and they have proven to be useful as beam splitters [24,25], grid polarizers [26,27,28,29,30,31], filters [32,33,34,35,36,37,38,39], Fabry–Perot interferometers [39,40,41,42,43], and output couplers for lasers [44,45,46,47,48,49,50,51].

A transversely corrugated cylindrical conducting rod was treated via a full-wave modal analytical method in [52,53], whereas a longitudinally corrugated counterpart was studied in [54] using the asymptotic corrugations boundary conditions (ACBCs). Core conducting rods sheathed by dielectric pipes whose outer surfaces were grated with transverse metallic strip gratings were analyzed via the asymptotic strips boundary conditions (ASBCs) in [55]. Shortly thereafter, the use of the ACBCs and coordinate transformation to treat parallel corrugated plates that were rotated from one another was reported in [56,57]. An analysis using the ASBCs to study transverse metal-strip-grated dielectric rods with a wide EBG for use as multifrequency cylindrical antennas was given in [58].

Over the past few years, there has been a surge in the amount of research on holographic antennas, and the underlying technique for the design of these is derived from the theory of holography [59,60,61]. Customarily in a planar form, the hologram is synthesized according to the interference pattern between the designated object waves and a known reference beam. When the interferogram is illuminated by this reference wave, the prescribed object waves are reconstructed. Studies of holographic antennas in less traditional cylindrical forms have also been reported [62,63,64,65], although in far smaller amounts.

**Figure 1:**
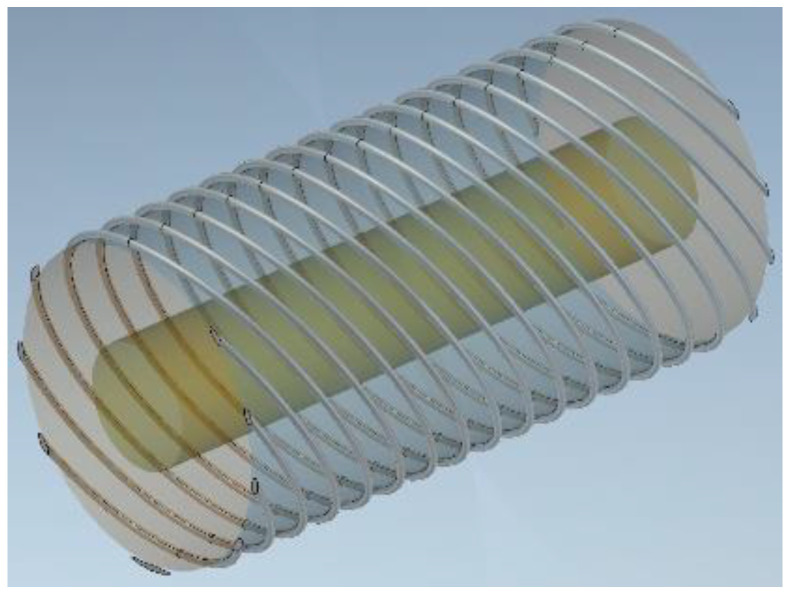
Perspective schematic view of skewed helical conducting strip-grating printed on outer surface of dielectric pipe wrapped over core dielectric rod.

In this paper, a dielectric rod with an exterior surface wound by an axially skewed helical metallic strip grating, as schematized in Figure 1, is studied using the ASBCs and the coordinate transformation technique. Aside from the inaugural use of these unique boundary conditions in the context of spiral gratings over a cylindrical surface, the chief innovation of the proposed approach is two-pronged. The first is the rotation of the typically straight axis coordinates on a planar surface, followed by the novel concept of rolling up the aforementioned flat, swiveled coordinate space to become a cylindrical surface. This results in one of the rolled-up rotated axes occurring along the spiral, thereby rendering it amenable to the ASBCs. Moreover, the classical modal technique of vector potentials is applied, which requires the grated direction to be along a coordinate axis such that the fields can be described in terms of it and the other orthogonal dimension, both tangential to the cylindrical surface. Interconnected sections of such metasurface rods, each with a certain skew angle pertaining to their distinct surface modal properties, enable the modulation of the surface impedance distribution over the cylindrical surface towards the synthesis of holographic rod antennas.

Such a helical structure constitutes one of the topologies among chiral media [66]; chirality is the property of asymmetry whereby an object cannot be superposed onto its mirror image, the earliest mention of which can be traced back to [67], where the term was first coined. This notion was subsequently further researched throughout the century, evolving into the topic of chiral metamaterials in recent years [68,69]. Applications derived from this phenomenon, natural or engineered, are essentially based on manipulated forms of optical activity, comprising birefringence and dichroism. Pertaining, respectively, to the different speeds and absorptions undergone by circularly polarized waves of opposite handedness as they pass through a chiral medium, the former property allows for rotations of linear polarization, while conversions between circular and linear ones can arise from the latter. Hence, the formulation presented herein for the treatment of this type of coil grating can be applied to extended investigations in research on chiral media.

As each such cylindrical structure with a certain skew-angle of its coil grating supports a bound surface mode with a distinct slow wave propagation constant that translates to an effective refractive index and thus a particular reactive surface impedance, connected series of such cylinders, all with the same core rod and outer pipe but just with differently-skewed gratings, enables the synthesis of cylindrical metasurface type holographic rod antennas [59,60,61,62,63,64,65].

The cylindrical holographic rod antenna presented herein is elegantly designed from first principles of holography theorem entailing surface reactive tensor elements, formulated rigorously under the framework of cylindrical coordinates, all based upon the modal solutions afforded by the ASBC analysis, as will be seen in Section 5 later. This is unlike prior works, such as [62], which upon considering a planar hologram without analysis of the cylindrical case, proceeded directly to the fabrication of the flat structure using a flexible material, followed simply by bending the soft prototype to become cylindrical in shape, thus lacking novelty and theoretical rigor. The approach in [63] adopted a planar mode approximation along with an optimization process to translate the conventional flat surface impedance to a curved one over the conformal cell, as well as segmentation of the cylindrical surface into several flat sections, treating each one in the conventional planar way, thus still falling short of an elegant analysis. The work of 64 turned to the partitioning of a shared conformal aperture into planar ones, and made use of coordinate projections and geometrical transformations from planar to conformal topologies, being a crude approach that lacks robustness as well. The work of [65] adopted the so-called method of major-axis matching to tie up the radius of the cylindrical metasurface cell to the major axis of the planar surface scalar impedance, thus also requiring a simplistic treatment of the more conventional planar configuration first.

After the geometrical and material details of the structure are described in the upcoming Section 2, mathematical details of the modal theory and analysis are given in Section 3. This is followed, in Section 4, by the validation of the results computed by a program code written based on the formulation with an independent commercial full-wave solver in terms of modal dispersion diagrams. Theory and formulation of the holographic rod antenna are presented in Section 5, computed results of which are given in Section 6. Measurements of manufactured prototypes are then reported in Section 7, after which the paper is concluded.

## 2. Description of the Structure

**Figure 2:**
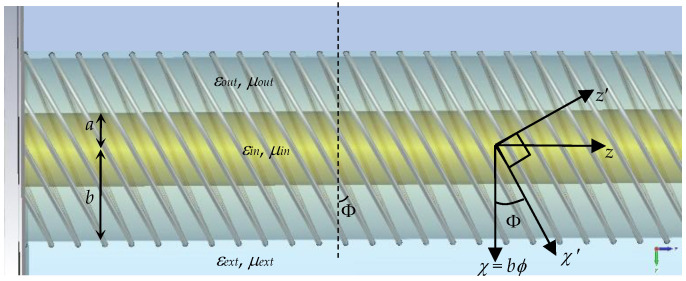
Lateral view of helical conducting strip-grating with skew angle Φ printed on outer surface of dielectric pipe with outer radius *b* and of medium (*ε_out_*, *μ_out_*) wrapped over core dielectric rod with radius *a* and of medium (*ε_in_*, *μ_in_*). Axes showing coordinate transformation as shown.

For a core rod of radius *a* wrapped by an outer pipe of outer radius *b*, the skewed helical metal strip grating wound over the outermost cylindrical surface with the latter radius is depicted in Figure 2. The material parameters of the rod and pipe are (*μ_in_*, *ε_in_*) and (*μ_out_*, *ε_out_*), respectively, while those of the external region are (*μ_ext_*, *ε_ext_*). Consider initially, a so-called unskewed *χz* coordinate plane as shown in the figure. Upon rolling this flat surface into a cylinder, just like rolling up a piece of paper, the *χ* = *bϕ* becomes the arc length associated with the usual *ϕ* in the cylindrical system while the *z* remains what it is. For conventional transversely strip-grated rods, this *bϕ* is then the coordinate along which the grating is aligned. Now, let this pair of *χz* flat coordinates be transformed, by rotating them through an angle of Φ, into a new primed *χ*′*z*′ likewise planar system, also as shown in Figure 2. Then, again by rolling up this coordinate-transformed flat *χ*′*z*′ plane into a cylindrical form, a spiral coordinate results, which can then be deemed as the curled-up axis along which the skewed-helical grating is oriented. This latter aspect then permits an analytical treatment by applying the ASBC to the outermost surface at *ρ* = *b* under the framework of this primed, transformed coordinate system. The skew is thus characterized by the tilt angle Φ made by the coil grating as seen from the viewed side of the structure with the transverse *ρϕ* plane, as illustrated in the schematic.

## 3. Theory and Formulation

### 3.1. Fields in Various Regions via Vector Potentials

The formulation beginning with the method of classical vector potentials may now ensue. The following universally applicable symbols are first defined:

(1) $$\Omega =\left\{\begin{array}{c} E\\ H \end{array}\right\};\;℧=\left\{\begin{array}{c} H\\ E \end{array}\right\};\;\zeta =\left\{\begin{array}{c} E\\ M \end{array}\right\};\;\upsilon =\left\{\begin{array}{c} \varepsilon \\ \mu \end{array}\right\};\;\prod =\left\{\begin{array}{c} F\\ A \end{array}\right\};\;C_{\phi }^{m}=\cos(m\phi );\;S_{\phi }^{m}=\sin(m\phi ),\;m\in \mathbb{Z}$$

In all upcoming relations, the items in the same kind of parenthesis (curly or triangular braces) correspond to one another throughout each equation.

The axial *z* components of the electric and magnetic vector potentials, *F_z_* and *A_z_*, for TE and TM modes (Tζ) respectively, may then be concisely expressed for all regions as

(2) $$\prod_{z_{T\zeta }}^{reg}=G_{\rho_{T\zeta },m}^{reg}\left(V_{\phi_{T\zeta }}^{reg}C_{\phi }^{m}+W_{\phi_{T\zeta }}^{reg}S_{\phi }^{m}\right)e^{-j\beta_{z_{T\zeta }}^{reg}z}$$

in which $V_{\phi_{T\zeta }}^{reg}$ and $W_{\phi_{T\zeta }}^{reg}$ are the initially unknown modal amplitude coefficients. Furthermore,

(3) $$\begin{array}{ll} G_{\rho_{T\zeta },m}^{reg}= & \left(1-\delta_{reg,ext}\right)\left[\left(V_{\rho_{T\zeta }}^{reg}-\delta_{reg,in}V_{\rho_{T\zeta }}^{reg}+\delta_{reg,in}\right)J_{m}(k_{\rho_{T\zeta }}^{reg}\rho )+\delta_{reg,out}W_{\rho_{T\zeta }}^{reg}Y_{m}(k_{\rho_{T\zeta }}^{reg}\rho )\right]\\ & +\;\delta_{reg,ext}H_{m}^{\left(2\right)}(k_{\rho_{T\zeta }}^{ext}\rho ) \end{array}$$

in which *J_m_* and *Y_m_* are the *m*th order Bessel functions of the first and second kinds respectively, $H_{m}^{\left(2\right)}$ is the *m*th order Hankel function of the second kind. The script *reg*, denoting “*region*”, may be either *in*, *out* or *ext*, and the delta symbol representing the Kronecker delta is defined as

(4) $$\delta_{p,q}=\left\{\begin{array}{c} 1,\;\mathrm{if}\;p=q\\ 0,\;\mathrm{if}\;p\neq q \end{array}\right.$$

The TE and TM modal phase constants *k_ρ_* and *β_z_* along the radial and axial coordinates of the various regions are signified by the *Tζ* and *reg* scripts attached to them. The various field components in all regions are then succinctly represented as follow.

(5) $$\Omega_{\rho_{T\zeta }}^{reg}=\left\{\mp \right\}\frac{1}{\upsilon_{reg}\rho }\frac{\partial }{\partial \phi }\prod_{z_{T\zeta }}^{reg}=\left\{\pm \right\}\frac{m}{\upsilon_{reg}\rho }G_{\rho_{T\zeta },m}^{reg}\left(V_{\phi_{T\zeta }}^{reg}S_{\phi }^{m}-W_{\phi_{T\zeta }}^{reg}C_{\phi }^{m}\right)e^{-j\beta_{z_{T\zeta }}^{reg}z}$$

(6) $$\Omega_{\phi_{T\zeta }}^{reg}=\left\{\pm \right\}\frac{1}{\upsilon_{reg}}\frac{\partial \prod_{z_{T\zeta }}^{reg}}{\partial \rho }=\left\{\pm \right\}\frac{k_{\rho_{T\zeta }}^{reg}}{\upsilon_{reg}}\frac{dG_{\rho_{T\zeta },m}^{reg}}{d\left(k_{\rho_{T\zeta }}^{reg}\rho \right)}\left(V_{\phi_{T\zeta }}^{reg}C_{\phi }^{m}+W_{\phi_{T\zeta }}^{reg}S_{\phi }^{m}\right)e^{-j\beta_{z_{T\zeta }}^{reg}z}$$

(7) $$\left[\begin{array}{c} {℧}_{\rho_{T\zeta }}^{reg}\\ {℧}_{\phi_{T\zeta }}^{reg}\\ {℧}_{z_{T\zeta }}^{reg} \end{array}\right]=\frac{1}{j\omega \mu_{reg}\varepsilon_{reg}}\left[\begin{array}{c} \frac{\partial^{2}}{\partial \rho \partial z}\\ \frac{1}{\rho }\frac{\partial^{2}}{\partial \phi \partial z}\\ \frac{\partial^{2}}{\partial z^{2}}+k_{reg}^{2} \end{array}\right]\prod_{z_{T\zeta }}^{reg}=\frac{e^{-j\beta_{z_{T\zeta }}^{reg}z}}{\omega \mu_{reg}\varepsilon_{reg}}\left[\begin{array}{c} -k_{\rho_{T\zeta }}^{reg}\beta_{z_{T\zeta }}^{reg}\frac{dG_{\rho_{T\zeta },m}^{reg}}{d\left(k_{\rho_{T\zeta }}^{reg}\rho \right)}\left(V_{\phi_{T\zeta }}^{reg}C_{\phi }^{m}+W_{\phi_{T\zeta }}^{reg}S_{\phi }^{m}\right)\\ m\beta_{z_{T\zeta }}^{reg}\rho^{-1}G_{\rho_{T\zeta },m}^{reg}\left(V_{\phi_{T\zeta }}^{reg}S_{\phi }^{m}-W_{\phi_{T\zeta }}^{reg}C_{\phi }^{m}\right)\\ -j\left[k_{reg}^{2}-{\left(\beta_{z_{T\zeta }}^{reg}\right)}^{2}\right]G_{\rho_{T\zeta },m}^{reg}\left(V_{\phi_{T\zeta }}^{reg}C_{\phi }^{m}+W_{\phi_{T\zeta }}^{reg}S_{\phi }^{m}\right) \end{array}\right]$$

The field components with respect to the primed *χ*′ and *z*′ coordinates are then written in terms of those under the unprimed system according to the following matrix equation:

(8) $$\left[\begin{array}{cc} \Omega_{{{\hat{\chi }}^{\prime }}_{T\zeta }}^{\langle out|ext\rangle } & {℧}_{{{\hat{\chi }}^{\prime }}_{T\zeta }}^{\langle out|ext\rangle }\\ \Omega_{{{\hat{z}}^{\prime }}_{T\zeta }}^{\langle out|ext\rangle } & {℧}_{{{\hat{z}}^{\prime }}_{T\zeta }}^{\langle out|ext\rangle } \end{array}\right]=\left[\begin{array}{cc} \cos\Phi & \sin\Phi \\ -\sin\Phi & \cos\Phi \end{array}\right]\left[\begin{array}{cc} \Omega_{\phi_{T\zeta }}^{\langle out|ext\rangle } & {℧}_{\phi_{T\zeta }}^{\langle out|ext\rangle }\\ 0 & {℧}_{z_{T\zeta }}^{\langle out|ext\rangle } \end{array}\right]$$

with one such matrix equation for each of the “*out*” and “*ext*” cases.

### 3.2. Boundary Conditions: Standard Ones and ASBC

The upcoming two relations express the standard boundary conditions (continuity of tangential fields) over the smooth ungrated interface between the inner core dielectric rod and outer dielectric pipe:

(9) $$\sum_{\Psi =E}^{\Psi =M}\Omega_{\phi_{T\Psi }}^{in}\left(\rho =a\right)=\sum_{\Psi =E}^{\Psi =M}\Omega_{\phi_{T\Psi }}^{out}\left(\rho =a\right)$$

(10) $${℧}_{z_{T\zeta }}^{in}\left(\rho =a\right)={℧}_{z_{T\zeta }}^{out}\left(\rho =a\right)$$

with one of these two equations for each of the two cases of Ω and ℧, respectively, as of (1).

Upon applying the ASBC to the helically grated interface between the outer dielectric pipe and exterior space, such that the *E*-field components parallel with the spiral grating vanish on both sides of it, whereas the *E* and *H* field components respectively perpendicular and parallel with it are continuous across the conducting helix, the following are obtained:

(11) $$\sum_{\Psi =E}^{\Psi =M}E_{{\chi^{\prime }}_{T\Psi }}^{\langle out|ext\rangle }\left(\rho =b\right)=0$$

with one such equation for “*out*” and “*ext*” cases, and

(12) $$\sum_{\Psi =E}^{\Psi =M}\Omega_{{{\hat{z}}^{\prime }}_{T\Psi }}^{out}\left(\rho =b\right)=\sum_{\Psi =E}^{\Psi =M}\Omega_{{{\hat{z}}^{\prime }}_{T\Psi }}^{ext}\left(\rho =b\right)$$

with one such equation for each of the two cases of Ω.

### 3.3. Matrix Equation

These latter boundary conditions, a total of 16 of them, may then be cast into a matrix equation with a system matrix ${\underline{M}}_{16\times 16}$ of size 16 × 16, as follows.

(13) $${\underline{M}}_{16\times 16}{\underline{C}}_{16\times 1}={\underline{0}}_{16\times 1}$$

where

(14) $${\underline{C}}_{16\times 1}={\left[\begin{array}{ccccc} C_{1} & C_{2} & \cdots & C_{15} & C_{16} \end{array}\right]}^{Τ}={\left[\begin{array}{c} {\left(\begin{array}{cccc} V_{\phi_{TE}}^{in} & W_{\phi_{TE}}^{in} & V_{\phi_{TM}}^{in} & W_{\phi_{TM}}^{in} \end{array}\right)}^{Τ}\\ {\left(\begin{array}{cccc} V_{\rho_{TE}}^{out}V_{\phi_{TE}}^{out} & V_{\rho_{TE}}^{out}W_{\phi_{TE}}^{out} & W_{\rho_{TE}}^{out}V_{\phi_{TE}}^{out} & W_{\rho_{TE}}^{out}W_{\phi_{TE}}^{out} \end{array}\right)}^{Τ}\\ {\left(\begin{array}{cccc} V_{\rho_{TM}}^{out}V_{\phi_{TM}}^{out} & V_{\rho_{TM}}^{out}W_{\phi_{TM}}^{out} & W_{\rho_{TM}}^{out}V_{\phi_{TM}}^{out} & W_{\rho_{TM}}^{out}W_{\phi_{TM}}^{out} \end{array}\right)}^{Τ}\\ {\left(\begin{array}{cccc} V_{\phi_{TE}}^{ext} & W_{\phi_{TE}}^{ext} & V_{\phi_{TM}}^{ext} & W_{\phi_{TM}}^{ext} \end{array}\right)}^{Τ} \end{array}\right]}_{16\times 1}$$

is the 16 × 1 column vector containing the modal amplitude coefficients. As for the matrix elements, by first defining the following representations:

$$\begin{array}{l} \bar{\alpha }=\langle \begin{array}{ccc} 1 & 1 & 1 \end{array}\rangle ;\;\bar{\beta }=\langle \begin{array}{ccc} 1 & 5 & 7 \end{array}\rangle ;\;\bar{\varsigma }=\langle \begin{array}{ccc} 1 & -1 & -1 \end{array}\rangle ;\;\bar{B}=\langle \begin{array}{ccc} J & J & Y \end{array}\rangle \\ \bar{\gamma }=\langle \begin{array}{ccc} in & out & out \end{array}\rangle ;\;\bar{\Delta }=\langle \begin{array}{ccc} 1 & 0 & 0 \end{array}\rangle ;\;\bar{\nabla }=\langle \begin{array}{ccc} 0 & 1 & 1 \end{array}\rangle \end{array}$$

(15) $$\begin{array}{l} \bar{□}=\langle \begin{array}{ccc} 0 & 1 & 0 \end{array}\rangle ;\;\bar{◊}=\langle \begin{array}{ccc} 1 & 1 & -1 \end{array}\rangle ;\;\\ \bar{\Gamma }=\langle \begin{array}{ccc} out & out & ext \end{array}\rangle ;\;\bar{N}=\langle \begin{array}{ccc} J & Y & H^{\left(2\right)} \end{array}\rangle \end{array}$$

(16) $${\bar{\Psi }}_{1}=\left\{\begin{array}{c} 1\\ \bar{◊}\\ 1 \end{array}\right\};\;{\bar{\Psi }}_{2}=\left\{\begin{array}{c} 1\\ -\bar{◊}\\ 1 \end{array}\right\};\;{\bar{\Psi }}_{3}=\left\{\begin{array}{c} -1\\ -\bar{◊}\\ 1 \end{array}\right\}$$

(17) $${\bar{\Psi }}_{1}=\left\{\begin{array}{c} \cos\Phi \\ \left(\sin\Phi \right)/\varepsilon_{\bar{\Gamma }}\\ -\bar{◊}\left(\cos\Phi \right)/\mu_{\bar{\Gamma }} \end{array}\right\};\;{\bar{\Psi }}_{2}=\left\{\begin{array}{c} \varepsilon_{\bar{\Gamma }}\cos\Phi \\ \cos\Phi \\ \bar{◊}\sin\Phi \end{array}\right\};\;{\bar{\Psi }}_{3}=\left\{\begin{array}{c} \varepsilon_{\bar{\Gamma }}\sin\Phi \\ -\cos\Phi \\ \bar{◊}\sin\Phi \end{array}\right\}$$

(18) $${\bar{\xi }}_{1}=\left\{\begin{array}{c} TE\\ TE\\ TM \end{array}\right\};\;{\bar{\xi }}_{2}=\left\{\begin{array}{c} TM\\ TE\\ TM \end{array}\right\};\;{\bar{\xi }}_{3}=\left\{\begin{array}{c} TM\\ TM\\ TE \end{array}\right\}$$

(19) $${\bar{ϒ}}_{〚\begin{array}{c} 1\\ 2 \end{array}〛}=\left\{\begin{array}{c} \left(\bar{\alpha }+8+〚\begin{array}{c} 0\\ 1 \end{array}〛\right)+\left(\bar{\alpha }-\bar{◊}\right),\bar{\beta }+4+〚\begin{array}{c} 0\\ 1 \end{array}〛-2\bar{\nabla }\bar{◊}\\ \bar{\alpha }+12+〚\begin{array}{c} 0\\ 1 \end{array}〛,4+〚\begin{array}{c} 0\\ 1 \end{array}〛+\bar{\beta }-2\bar{◊}\bar{\nabla }\\ \bar{\alpha }+14+〚\begin{array}{c} 0\\ 1 \end{array}〛,\bar{\beta }+8+〚\begin{array}{c} 0\\ 1 \end{array}〛-2\bar{□} \end{array}\;\right\}$$

(20) $${\bar{ϒ}}_{〚\begin{array}{c} 3\\ 4 \end{array}〛}=\left\{\begin{array}{c} \left(\bar{\alpha }+8+〚\begin{array}{c} 0\\ 1 \end{array}〛\right)+\left(\bar{\alpha }-\bar{◊}\right),\bar{\beta }+9-〚\begin{array}{c} 0\\ 1 \end{array}〛-2\bar{□}\\ \bar{\alpha }+14+〚\begin{array}{c} 0\\ 1 \end{array}〛,\bar{\beta }+5-〚\begin{array}{c} 0\\ 1 \end{array}〛-2\bar{◊}\bar{\nabla }\\ \bar{\alpha }+12+〚\begin{array}{c} 0\\ 1 \end{array}〛,\bar{\beta }+9-〚\begin{array}{c} 0\\ 1 \end{array}〛-2\bar{□} \end{array}\;\right\}$$

(21) $${\bar{ϒ}}_{〚\begin{array}{c} 5\\ 6 \end{array}〛}=\left\{\begin{array}{c} \left(\bar{\alpha }+8+〚\begin{array}{c} 0\\ 1 \end{array}〛\right)+\left(\bar{\alpha }-\bar{◊}\right),\bar{\beta }+8+〚\begin{array}{c} 0\\ 1 \end{array}〛-2\bar{□}\\ \bar{\alpha }+12+〚\begin{array}{c} 0\\ 1 \end{array}〛,8+〚\begin{array}{c} 0\\ 1 \end{array}〛+\bar{\beta }-2\bar{□}\\ \bar{\alpha }+14+〚\begin{array}{c} 0\\ 1 \end{array}〛,\bar{\beta }+4+〚\begin{array}{c} 0\\ 1 \end{array}〛-2\bar{◊}\bar{\nabla } \end{array}\;\right\}$$

the elements of ${\underline{M}}_{16\times 16}$ may be expressed compactly as follow (matrix locations not specified contain zeros).

(22) $$M_{\left\{\begin{array}{c} \bar{\alpha },\bar{\beta }\\ \bar{\alpha }+4,\bar{\beta }+2\bar{\Delta }+4\bar{\nabla } \end{array}\right\}}=M_{\left\{\begin{array}{c} \bar{\alpha }+1,\bar{\beta }+1\\ \bar{\alpha }+5,\bar{\beta }+2\bar{\Delta }+4\bar{\nabla }+1 \end{array}\right\}}=\left\{\pm \right\}\bar{\varsigma }\frac{k_{\rho_{T\zeta }}^{\bar{\gamma }}}{\upsilon_{\bar{\gamma }}}{{\bar{B}}^{\prime }}_{m}(k_{\rho_{T\zeta }}^{\bar{\gamma }}a)$$

(23) $$M_{\left\{\begin{array}{l} \bar{\alpha }+\bar{\Delta }+4,\bar{\beta }+\bar{\nabla }\\ \bar{\alpha }+\bar{\Delta },\bar{\beta }+2\bar{\Delta }+5\bar{\nabla } \end{array}\right\}}=-M_{\left\{\begin{array}{l} \bar{\alpha }+\bar{\Delta }+4-\bar{\varsigma },\bar{\beta }+\bar{\nabla }+\bar{\varsigma }\\ \bar{\alpha }+\bar{\Delta }-\bar{\varsigma },\bar{\beta }+2\bar{\Delta }+5\bar{\nabla }+\bar{\varsigma } \end{array}\right\}}=\frac{m\beta_{z_{T\zeta }}^{\bar{\gamma }}}{\omega \mu_{\bar{\gamma }}\varepsilon_{\bar{\gamma }}a}{\bar{B}}_{m}(k_{\rho_{T\zeta }}^{\bar{\gamma }}a)$$

(24) $$M_{\left\{\begin{array}{c} 7\bar{\alpha },\bar{\beta }\\ 3\bar{\alpha },\bar{\beta }+2\bar{\Delta }+4\bar{\nabla } \end{array}\right\}}=M_{\left\{\begin{array}{c} 7\bar{\alpha }+1,\bar{\beta }+1\\ 3\bar{\alpha }+1,\bar{\beta }+2\bar{\Delta }+4\bar{\nabla }+1 \end{array}\right\}}=\left\{\mp \right\}\bar{\varsigma }\frac{k_{\bar{\gamma }}^{2}-{\left(\beta_{z_{T\zeta }}^{\bar{\gamma }}\right)}^{2}}{\mu_{\bar{\gamma }}\varepsilon_{\bar{\gamma }}}{\bar{B}}_{m}(k_{\rho_{T\zeta }}^{\bar{\gamma }}a)$$

(25) $${\bar{\Psi }}_{2}M_{{\bar{ϒ}}_{1}}={\bar{\Psi }}_{1}M_{{\bar{ϒ}}_{2}}={\bar{\Psi }}_{1}k_{\rho_{{\bar{\xi }}_{1}}}^{\bar{\Gamma }}{{\bar{N}}^{\prime }}_{m}(k_{\rho_{{\bar{\xi }}_{1}}}^{\bar{\Gamma }}b)$$

(26) $${\bar{\Psi }}_{3}M_{{\bar{ϒ}}_{3}}={\bar{\Psi }}_{1}M_{{\bar{ϒ}}_{4}}={\bar{\Psi }}_{2}\frac{m\beta_{z_{{\bar{\xi }}_{2}}}^{\bar{\Gamma }}{\bar{N}}_{m}(k_{\rho_{{\bar{\xi }}_{2}}}^{\bar{\Gamma }}b)}{\omega \mu_{\bar{\Gamma }}\varepsilon_{\bar{\Gamma }}b}$$

(27) $${\bar{\Psi }}_{2}M_{{\bar{ϒ}}_{5}}={\bar{\Psi }}_{1}M_{{\bar{ϒ}}_{6}}={\bar{\Psi }}_{3}\frac{k_{\bar{\Gamma }}^{2}-{\left(\beta_{z_{{\bar{\xi }}_{3}}}^{\bar{\Gamma }}\right)}^{2}}{j\omega \mu_{\bar{\Gamma }}\varepsilon_{\bar{\Gamma }}}{\bar{N}}_{m}(k_{\rho_{{\bar{\xi }}_{3}}}^{\bar{\Gamma }}b)$$

in all of which, the items within each kind of braces in any one relation correspond to one another throughout the equations, independently of those within the other types of braces. The primes attached to *B* and *N* symbolizing a Bessel or Hankel function denote the derivatives of them with respect to their arguments, being $k_{\rho_{T\zeta }}^{\bar{\gamma }}\rho$ or $k_{\rho_{{\bar{\xi }}_{\#}}}^{\bar{\Gamma }}\rho$, and then evaluated at $k_{\rho_{T\zeta }}^{\bar{\gamma }}a$ or $k_{\rho_{{\bar{\xi }}_{\#}}}^{\bar{\Gamma }}\rho$.

The universal axial propagation phase constant *β_z_^univ^* and the wavenumber component $k_{\rho_{T\zeta }}^{reg}$ along the radial *ρ* coordinate are related according to

(28) $${\left(k_{\rho_{T\zeta }}^{reg}\right)}^{2}+{\left(\beta_{z}^{univ}\right)}^{2}=k_{reg}^{2}=\omega^{2}\mu_{reg}\varepsilon_{reg}$$

With $\Delta (\beta_{z}^{univ},f)=\det\left({\underline{M}}_{16\times 16}\right)$ signifying the determinant of the system matrix, which is a function of the universal axial propagation phase constant *β_z_^univ^* = $\beta_{z_{T\zeta }}^{reg}$ and the frequency *f*, the characteristic equation is then expressed as:

(29) $$\Delta (\beta_{z,reson}^{univ},f_{reson})\overset{equated}{\underset{to}{=}}0$$

which is satisfied by the coordinate pair: $\left(\beta_{z,reson}^{univ},f_{reson}\right)$, being the roots of the equation and constituting the eigen-modal resonance which may be determined numerically (e.g., by sweeping through a search space over both coordinate variables/parameters to detect modal resonances). These detected eigen-modal resonance conditions are then substituted into the expressions for the matrix elements of ${\underline{M}}_{16\times 16}$ to yield the resonant ${\left.{\underline{M}}_{16\times 16}^{reson}\right|}_{f_{res},\beta_{res}}$ for those resonant coordinates. This latter may then be transformed into its row-echelon form (upper-triangular matrix) by Gauss elimination, which upon back substitution, yields the eigen-vector ${\underline{C}}_{16\times 1}$ of (14) containing the unknown amplitude coefficients, all scaled by an arbitrary coefficient, typically the last element *C*_16_ in it.

### 3.4. Far-Field Radiation

By classical aperture theory, the tangential electric and magnetic fields on the outermost cylindrical surface of the rod structure relate, via the surface equivalence theorem, to equivalent cylindrical aperture magnetic and electric current densities, $\vec{J}({\vec{r}}^{\prime })$ and $\vec{M}({\vec{r}}^{\prime })$, that are responsible for the far-field radiation.

With a *ρ*-level of the aperture taken to be *ρ*′ = *b*, the far-fields radiated by these latter $\vec{J}({\vec{r}}^{\prime })$ and $\vec{M}({\vec{r}}^{\prime })$ are stated as follow [70]. Starting with the radiated E-field,

(30) $$\vec{E}(\hat{r})=\frac{e^{-jkr}}{r}\left[{\vec{G}}_{J}(\hat{r})+{\vec{G}}_{M}(\hat{r})\right]$$

where $\hat{r}(\theta ,\phi )=\hat{x}\sin\theta \cos\phi +\hat{y}\sin\theta \sin\phi +\hat{z}\cos\theta$ is the radial unit vector of the spherical coordinate system pointing in the direction (*θ*, *ϕ*) of far-field observation, and

(31) $${\vec{G}}_{J}(\hat{r})={\vec{I}}_{J}-\left({\vec{I}}_{J}\cdot \hat{r}\right)\hat{r}$$

(32) $${\vec{G}}_{M}(\hat{r})={\vec{I}}_{M}\times \hat{r}$$

(33) $${\vec{I}}_{J}=-\frac{jk\eta }{4\pi }\int_{z^{\prime }=z_{1}}^{z^{\prime }=z_{2}}\int_{\phi^{\prime }=0}^{\phi^{\prime }=2\pi }\vec{J}({\vec{r}}^{\prime })e^{jk\left({\vec{r}}^{\prime }\cdot \hat{r}\right)}{\rho^{\prime }}_{a}d\phi^{\prime }dz^{\prime }$$

(34) $${\vec{I}}_{M}=-\frac{jk}{4\pi }\int_{z^{\prime }=z_{1}}^{z^{\prime }=z_{2}}\int_{\phi^{\prime }=0}^{\phi^{\prime }=2\pi }\vec{M}({\vec{r}}^{\prime })e^{jk\left({\vec{r}}^{\prime }\cdot \hat{r}\right)}{\rho^{\prime }}_{a}d\phi^{\prime }dz^{\prime }$$

in which ${\vec{r}}^{\prime }$ is the position (radial) vector of the source point, thus being a function of the source coordinates *ϕ*′ and *z*′, which are the integration variables, and where *k* = 2*πf*√(*μ_ext_ε_ext_*) and *η* = √(*μ_ext_*/*ε_ext_*). The length of the grated rod is thus *L* = *z*_2_ − *z*_1_. The cylindrical aperture currents are written as

(35) $$\vec{J}({\vec{r}}^{\prime })=\hat{\rho }\times {\vec{H}}^{ext}({\rho^{\prime }}_{a})$$

(36) $$\vec{M}({\vec{r}}^{\prime })={\vec{E}}^{ext}({\rho^{\prime }}_{a})\times \hat{\rho }$$

where ${\vec{E}}^{ext}$ and ${\vec{H}}^{ext}$ are those of Section 3.1.

The corresponding radiated *H*-field is given by

(37) $$\vec{H}(\hat{r})=(\eta^{-1})\hat{r}\times \vec{E}(\hat{r})$$

## 4. Validation with Independent Solver

Generated by program codes developed according to the modal approach of Section 3, this section presents results of modal dispersion diagrams for skewed helical strip-grated dielectric pipes wrapped over core dielectric rods, and validated with those simulated by a commercial simulation software solver: CST Microwave Studio 2019 (henceforth just CST).

**Figure 3:**
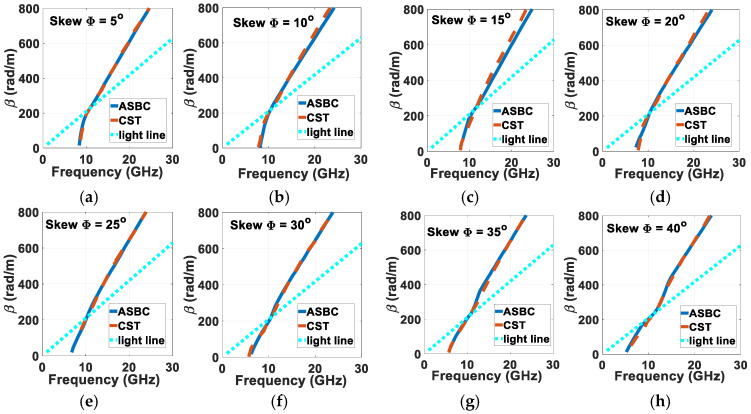
Modal dispersion diagrams for *m* = 1, *a* = 3 mm, *b* = 6 mm, (*μ_in_*, *ε_in_*) = (*μ*_0_, 2.2*ε*_0_), (*μ_out_*, *ε_out_*) = (*μ*_0_, 3.8*ε*_0_), (*μ_ext_*, *ε_ext_*) = (*μ*_0_, *ε*_0_), computed by presented ASBC-based analysis and simulated by CST, for (**a**) Φ = 5°, (**b**) Φ = 10°, (**c**) Φ = 15°, (**d**) Φ = 20°, (**e**) Φ = 25°, (**f**) Φ = 30°, (**g**) Φ = 35°, (**h**) Φ = 40°.

Upon solving (29) for the eigen-modal coordinates $\left(\beta_{z,reson}^{univ},f_{reson}\right)$ via the roots of the equation, these resonant quantities may be plotted against each other to produce the modal dispersion diagram. Doing so for an arbitrary set of parameters: *a* = 3 mm, *b* = 6 mm, (*μ_in_*, *ε_in_*) = (*μ*_0_, 2.2*ε*_0_), (*μ_out_*, *ε_out_*) = (*μ*_0_, 3.8*ε*_0_), (*μ_ext_*, *ε_ext_*) = (*μ*_0_, *ε*_0_), the dispersion graphs of $\beta_{z,reson}^{univ}$ vs. *f_reson_* for *m* = 1 generated by the herein presented formulation for skew angles Φ from 5° to 40° in steps of 5° are presented in Figure 3a–h in that respective order. Accompanied with these in the same plots are corresponding traces simulated by CST. Good agreement between both tools is observed.

**Figure 4:**
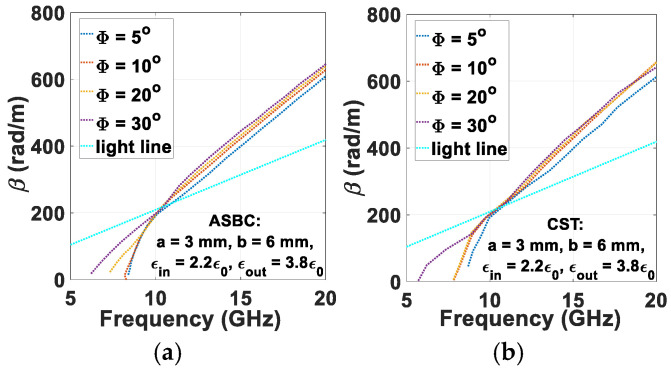
Modal dispersion diagrams for *m* = 1, *a* = 3 mm, *b* = 6 mm, (*μ_in_*, *ε_in_*) = (*μ*_0_, 2.2*ε*_0_), (*μ_out_*, *ε_out_*) = (*μ*_0_, 3.8*ε*_0_), (*μ_ext_*, *ε_ext_*) = (*μ*_0_, *ε*_0_), for various Φ (5°, 10°, 20°, and 30°), (**a**) computed by code according to ASBC-based analysis, and (**b**) simulated by CST.

Plotted in Figure 4a,b are the dispersion curves for various skew angles as indicated in the legends, each graph obtained by the same aforementioned tools, respectively being the computer code written according to the ASBC-based analysis and the CST software. The upward moving trend of the traces with increasing Φ is demonstrated by both solution approaches.

**Figure 5:**
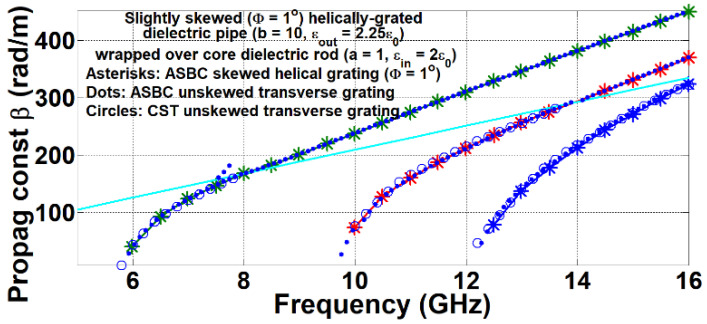
Modal dispersion diagrams for *a* = 1 mm, *b* = 10 mm, (*μ_in_*, *ε_in_*) = (*μ*_0_, 2*ε*_0_), (*μ_out_*, *ε_out_*) = (*μ*_0_, 2.25*ε*_0_), (*μ_ext_*, *ε_ext_*) = (*μ*_0_, *ε*_0_), Φ = 1°, computed by presented ASBC-based analysis (asterisk markers) and by likewise ASBC-based method for treating corresponding conventional transverse circumferential metal circular strip grated rod (dot markers), as well as simulated by CST (circle markers).

When the skew angle Φ is very small, the spiral grating becomes an essentially unskewed version (like a highly compressed coil spring) and approaches the conventional transverse strip grated rod, i.e., circumferential metallic circular strip coils. For another randomly prescribed set of parameters: *a* = 1 mm, *b* = 10 mm, (*μ_in_*, *ε_in_*) = (*μ*_0_, 2*ε*_0_), (*μ_out_*, *ε_out_*) = (*μ*_0_, 2.25*ε*_0_), (*μ_ext_*, *ε_ext_*) = (*μ*_0_, *ε*_0_), and a small skew angle of Φ = 1°, the modal dispersion curves computed by the formulated method are conveyed by asterisk markers in Figure 5. Presented in the same graph are modal traces obtained by a likewise ASBC-based vector-potential analytical approach [58] for the corresponding transverse strip-grated rods and represented by dot markers, as well as those simulated by CST given as circle markers.

## 5. Holographic Rod Antenna

### 5.1. Surface Impedance Tensor Fundamentals

On a tensor impedance surface, the surface wave modes are in general neither pure TM nor TE, but rather, a hybrid. As such, the total surface fields are written as

(38) $${\vec{\Omega }}_{surf}^{TOT}={\left[\begin{array}{ccc} \Omega_{\rho }^{TOT}=0 & \Omega_{\phi }^{TOT} & \Omega_{z}^{TOT} \end{array}\right]}^{Τ}={\vec{\Omega }}_{surf}^{TM}+℘{\vec{\Omega }}_{surf}^{TE}$$

where ℘ is the arbitrary ratio of the TE modal amplitude to that of the TM mode and the superscript T denotes non-conjugate transposition. With the tensor impedance matrix $\underline{Z}$ and surface electric current $\vec{J}$ respectively expressed by

(39) $$\underline{Z}=\left[\begin{array}{cc} Z_{\phi \phi } & Z_{\phi z}\\ Z_{z\phi } & Z_{zz} \end{array}\right]$$

(40) $$\vec{J}=\hat{\rho }\times {\vec{H}}_{surf}^{TOT}={\left[\begin{array}{ccc} J_{\rho }=0 & J_{\phi }=-H_{z}^{TOT} & J_{z}=H_{\phi }^{TOT} \end{array}\right]}^{Τ}$$

the tensor impedance boundary condition on the cylindrical surface (all evaluated at *ρ* = *b*), expressed by a matrix equation relating the surface *E*-fields to the surface electric *J* currents via the impedance tensor $\underline{Z}$, is stated as:

(41) $${\vec{E}}_{surf}^{TOT}=\underline{Z}\cdot \vec{J}=\hat{\phi }\left(-Z_{\phi \phi }H_{z}^{TOT}+Z_{\phi z}H_{\phi }^{TOT}\right)+\hat{z}\left(-Z_{z\phi }H_{z}^{TOT}+Z_{zz}H_{\phi }^{TOT}\right)$$

Using the analytical modal field solutions of Section 3, the TM and TE modal surface fields at *ρ* = *b* are written as follow.

(42) $$\langle \begin{array}{c} {\vec{\Omega }}_{surf}^{T\zeta }\\ {\vec{℧}}_{surf}^{T\zeta } \end{array}\rangle =\langle \begin{array}{c} \hat{\phi }\Omega_{\phi_{T\zeta }}^{ext}\\ \hat{z}{℧}_{z_{T\zeta }}^{ext} \end{array}\rangle =\langle \begin{array}{c} \left\{\pm \right\}\hat{\phi }\frac{k_{\rho_{T\zeta }}^{ext}}{\upsilon_{ext}}\left\{\begin{array}{c} ℵ(m\phi )\\ 1 \end{array}\right\}{H_{m}^{\left(2\right)}}^{\prime }(k_{\rho_{T\zeta }}^{ext}b)\\ \hat{z}\frac{k_{ext}^{2}-{\left(\beta_{z_{T\zeta }}^{ext}\right)}^{2}}{j\omega \mu_{ext}\varepsilon_{ext}}\left\{\begin{array}{c} ℵ(m\phi )\\ 1 \end{array}\right\}H_{m}^{\left(2\right)}(k_{\rho_{T\zeta }}^{ext}b) \end{array}\rangle \Xi (\phi ,z)$$

(43) $$ℵ(m\phi )=\frac{V_{\phi_{TE}}^{ext}C_{\phi }^{m}+W_{\phi_{TE}}^{ext}S_{\phi }^{m}}{V_{\phi_{TM}}^{ext}C_{\phi }^{m}+W_{\phi_{TM}}^{ext}S_{\phi }^{m}}$$

(44) $$\Xi (\phi ,z)=\left(V_{\phi_{TM}}^{ext}C_{\phi }^{m}+W_{\phi_{TM}}^{ext}S_{\phi }^{m}\right)e^{-j\beta_{z_{TE}}^{ext}z}$$

with $k_{\rho_{T\zeta }}^{ext}=-j\alpha_{\rho }$ and $k_{ext}^{2}-{\left(\beta_{z_{T\zeta }}^{ext}\right)}^{2}=-\alpha_{\rho }^{2}$ for surface wave modes, *α_ρ_* being the positive real-valued attenuation constant along the radial direction. As before, the items within each kind of braces (curly or triangular braces) in any one relation correspond to one another throughout the equations, independently of those within the other types of braces, and (1) is again used.

The *ϕ* and *z* components of (41) then respectively lead to

(45) $$\frac{ℵ(m\phi )}{\varepsilon_{ext}}\left[Z_{\phi \phi }\frac{\alpha_{\rho }^{2}}{\omega \mu_{ext}}H_{m}^{\left(2\right)}(-j\alpha_{\rho }b)-\alpha_{\rho }{H_{m}^{\left(2\right)}}^{\prime }(-j\alpha_{\rho }b)\right]=Z_{\phi z}\frac{\alpha_{\rho }}{\mu_{ext}}{H_{m}^{\left(2\right)}}^{\prime }(-j\alpha_{\rho }b)$$

(46) $$Z_{zz}{H_{m}^{\left(2\right)}}^{\prime }(-j\alpha_{\rho }b)=\frac{\alpha_{\rho }}{\omega \varepsilon_{ext}}H_{m}^{\left(2\right)}(-j\alpha_{\rho }b)\left[1+ℵ(m\phi )Z_{z\phi }\right]$$

Dividing (45) by (46) thereby canceling out ℵ(mϕ) followed by lengthy albeit straightforward algebraic working, we obtain

(47) $$\begin{array}{l} Z_{\phi \phi }{\left[H_{m}^{\left(2\right)}(-j\alpha_{\rho }b)\right]}^{2}{\left(\alpha_{\rho }/k_{ext}\right)}^{2}\\ +H_{m}^{\left(2\right)}(-j\alpha_{\rho }b){H_{m}^{\left(2\right)}}^{\prime }(-j\alpha_{\rho }b)\left(Z_{\phi z}Z_{z\phi }\eta_{ext}^{-1}-Z_{\phi \phi }Z_{zz}\eta_{ext}^{-1}-\eta_{ext}\right)\frac{\alpha_{\rho }}{k_{ext}}+Z_{zz}{\left[{H_{m}^{\left(2\right)}}^{\prime }(-j\alpha_{\rho }b)\right]}^{2}=0 \end{array}$$

Solution of this quadratic equation leads to

(48) $$\left[H_{m}^{\left(2\right)}(-j\alpha_{\rho }b)/{H_{m}^{\left(2\right)}}^{\prime }(-j\alpha_{\rho }b)\right]\eta_{ext}\sqrt{n_{eff}^{2}-1}=\frac{\left\{\begin{array}{l} -\left(Z_{\phi z}Z_{z\phi }-Z_{\phi \phi }Z_{zz}-\eta_{ext}^{2}\right)\\ \pm \sqrt{{\left[\left(Z_{\phi z}Z_{z\phi }-Z_{\phi \phi }Z_{zz}-\eta_{ext}^{2}\right)\right]}^{2}-4Z_{\phi \phi }Z_{zz}\eta_{ext}^{2}} \end{array}\right\}}{2Z_{\phi \phi }}$$

where *n_eff_* = *k_z_*/*k*_0_ is the effective refractive index, with $k_{z}=\beta_{z,reson}^{univ}$, and *η_ext_* = √(*μ_ext_*/*ε_ext_*).

From the modal field solutions provided in Section 3, the ratio of the TM modal *E_z_* to *H_ϕ_* field components of the exterior (free space) region evaluated at the outer surface (*ρ* = *b*) constituting the cylindrical surface impedance is written as

(49) $$Z_{TM}=\frac{E_{z_{TM}}^{ext}(\rho =b)}{H_{\phi_{TM}}^{ext}(\rho =b)}=\frac{jk_{\rho }^{ext}}{\omega \varepsilon_{ext}}\frac{H_{m}^{\left(2\right)}(k_{\rho }^{ext}b)}{{H_{m}^{\left(2\right)}}^{\prime }(k_{\rho }^{ext}b)}$$

Under slow surface wave modal propagation along the cylindrical surface, the wavenumber along the radial *ρ* direction is purely negative imaginary, i.e., $k_{\rho }^{ext}=-j\alpha_{\rho }^{ext}$, with

(50) $$\alpha_{\rho }^{ext}=\sqrt{k_{z}^{2}-k_{0}^{2}}=k_{0}\sqrt{n_{eff}^{2}-1},\;k_{z}=\beta_{z,reson}^{univ}$$

Substituting this (50) into (49), and with (*μ_ext_*, *ε_ext_*) = (*μ*_0_, *ε*_0_) and *η*_0_ = √(*μ*_0_/*ε*_0_), we obtain

(51) $$Z_{TM}=\frac{H_{m}^{\left(2\right)}(-jk_{0}b\sqrt{n_{eff}^{2}-1})}{{H_{m}^{\left(2\right)}}^{\prime }(-jk_{0}b\sqrt{n_{eff}^{2}-1})}\eta_{0}\sqrt{n_{eff}^{2}-1}$$

which is then seen to be the left-hand side (LHS) of (48).

### 5.2. Holography Theorem

For an object wave of *E*-field ${\vec{E}}_{obj}$ and a reference wave of electric current ${\vec{J}}_{ref}=\hat{\rho }\times {\vec{H}}_{ref}$ manifested by the associated *H*-field ${\vec{H}}_{ref}$ of the reference wave, i.e.,

(52) $${\vec{J}}^{ref}=\hat{\rho }\times {\vec{H}}^{ref}={\left[\begin{array}{ccc} J_{\rho }^{ref}=0 & J_{\phi }^{ref}=-H_{z}^{ref} & J_{z}^{ref}=H_{\phi }^{ref} \end{array}\right]}^{Τ}$$

the interference pattern $\underline{\Pi }$ is written as

(53) $$\underline{\Pi }={\vec{E}}_{obj}⊗{\vec{J}}_{ref}^{†}=\left[\begin{array}{c} E_{\phi }^{obj}\\ E_{z}^{obj} \end{array}\right]⊗{\left[\begin{array}{c} J_{\phi }^{ref}\\ J_{z}^{ref} \end{array}\right]}^{†}=\left[\begin{array}{cc} -E_{\phi }^{obj}H_{z}^{ref*} & E_{\phi }^{obj}H_{\phi }^{ref*}\\ -E_{z}^{obj}H_{z}^{ref*} & E_{z}^{obj}H_{\phi }^{ref*} \end{array}\right]$$

in which ⊗ symbolizes the tensor or outer product and the † superscript denotes the Hermitian conjugate (conjugated transposition).

The form of this (53) is such that:

(54) $$\underline{\Pi }\cdot {\vec{J}}_{ref}={\left|{\vec{J}}_{ref}\right|}^{2}{\vec{E}}_{obj}$$

thus fully recovering the object field ${\vec{E}}_{obj}$.

Comparing this (54) with (41), it is readily recognized that the interference pattern $\underline{\Pi }$ matrix bears the same significance as the impedance tensor matrix $\underline{Z}$ of (39), i.e.,

(55) $$\underline{\Pi }=\left[\begin{array}{cc} -E_{\phi }^{obj}H_{z}^{ref*} & E_{\phi }^{obj}H_{\phi }^{ref*}\\ -E_{z}^{obj}H_{z}^{ref*} & E_{z}^{obj}H_{\phi }^{ref*} \end{array}\right]=\underline{Z}=\left[\begin{array}{cc} Z_{\phi \phi } & Z_{\phi z}\\ Z_{z\phi } & Z_{zz} \end{array}\right]$$

in which the reference *H_ref_* field components are from (42), being the surface magnetic fields, and the *E_obj_* terms are those of the known object wave.

With lower-cased *e* and *h* subscripts denoting TE and TM modes respectively, the object wave ${\vec{E}}^{obj}$ field is expressed by

(56) $${\vec{E}}^{obj}=\left[\begin{array}{c} E_{\rho }^{obj}=E_{\theta_{0m}}\Psi_{0m}\cos\theta_{0m}\\ E_{\phi }^{obj}=E_{\phi_{0e}}\Psi_{0e}\\ E_{z}^{obj}=-E_{\theta_{0m}}\Psi_{0m}\sin\theta_{0m} \end{array}\right]$$

(57) $$\Psi_{0{\{}_{e}^{m}\}}=\exp\left[-j\left(k_{0}\cos\theta_{0{\{}_{e}^{m}\}}\right)z\right]$$

with $E_{\theta_{0m}}$ and $E_{\phi_{0e}}$ being arbitrary amplitude coefficients and *θ*_0*m*_ and *θ*_0*e*_ are the angles of the object waves, being the usual elevation angles with respect to the axial *z* axis of the rod.

With the ${\vec{H}}_{surf}^{TM}=\hat{\phi }H_{\phi_{TM}}^{ext}$ and ${\vec{H}}_{surf}^{TE}=\hat{z}H_{z_{TE}}^{ext}$ of (42) now deemed respectively as $\hat{\phi }H_{\phi }^{ref}=\hat{\phi }J_{z}^{ref}$ and $\hat{z}H_{z}^{ref}=-\hat{z}J_{\phi }^{ref}$, the elements of the impedance tensor matrix in (39) as manifested by those of the interference pattern in (53) are obtained.

All $k_{\rho_{T\zeta }}^{ext}=-j\alpha_{\rho_{T\zeta }}^{ext}=-jk_{0}\sqrt{n_{eff}^{2}-1}$ are functions of the effective refractive index *n_eff_*. In addition, all surface wavenumbers $\beta_{z_{T\zeta }}^{ext}=k_{ext}n_{eff}>k_{ext}$ are also dependent on that same *n_eff_*. Furthermore, for a certain *m* circumferential harmonic and a particular frequency, since each skew angle (of the grating) pertains to a certain solved eigen-modal surface wavenumber $\beta_{z_{T\zeta }}^{ext}=k_{ext}n_{eff}$ as well as a set of the three solved modal amplitude coefficients of the exterior region: $V_{\phi_{TE}}^{ext}$, $W_{\phi_{TE}}^{ext}$, and $V_{\phi_{TM}}^{ext}$ (excluding $W_{\phi_{TM}}^{ext}$, which is the last element *C*_16_ of the eigenvector that is set to unity in the Gauss elimination followed by back-substitution in solving for the eigenvector, as mentioned earlier, and is thus fixed throughout), each of these coefficients is therefore a function of $\beta_{z_{T\zeta }}^{ext}=k_{ext}n_{eff}$. Consequently, each coefficient is also a separate function of the skew angle Φ and the effective refractive index *n_eff_*, i.e., for every $\beta_{z_{T\zeta }}^{ext}=k_{ext}n_{eff}$, there corresponds a certain coefficient $V_{\phi_{TE}}^{ext}$, $W_{\phi_{TE}}^{ext}$, or $V_{\phi_{TM}}^{ext}$. As such, for a string of skew angles with its associated string of solved $\beta_{z_{T\zeta }}^{ext}=k_{ext}n_{eff}$, there corresponds a string of coefficient values: $V_{\phi_{TE}}^{ext}$, $W_{\phi_{TE}}^{ext}$, and $V_{\phi_{TM}}^{ext}$. Each of these coefficients, being a numerical function of $\beta_{z_{T\zeta }}^{ext}=k_{ext}n_{eff}$ and thus *n_eff_*, may then be, by polynomial curve-fitting, converted to an analytical closed-form polynomial function of *n_eff_*. In other words, we obtain

(58) $$\mathbb{C}(n_{eff})=P_{N}n_{eff}^{N}+P_{N-1}n_{eff}^{N-1}+\cdots +P_{1}n_{eff}+P_{0}$$

where ℂ is either $V_{\phi_{TE}}^{ext},W_{\phi_{TE}}^{ext}$, or $V_{\phi_{TM}}^{ext}$.

**Figure 6:**
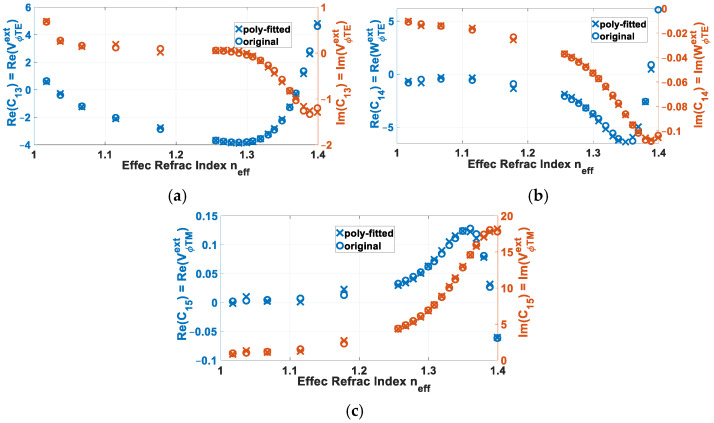
Real and imaginary parts of eigenvector coefficients: (**a**) *C*_13_, (**b**) *C*_14_, and (**c**) *C*_15_, plotted versus effective refractive index *n_eff_* = *β_z_^univ^*/*k*_0_, each pertaining to a Φ. Original solved ones of (14) given by circle markers, and reconstructed by polynomial curve-fitting with degree *N* = 6 (crosses), as of (58), for *m* = 1, *f_reson_* = 14 GHz, *a* = 3 mm, *b* = 6 mm, (*μ_in_*, *ε_in_*) = (*μ*_0_, 2.2*ε*_0_), (*μ_out_*, *ε_out_*) = (*μ*_0_, 3.8*ε*_0_), (*μ_ext_*, *ε_ext_*) = (*μ*_0_, *ε*_0_).

This is showcased by Figure 6, for polynomial degree of *N* = 6 and at a frequency of 14 GHz as well as the same previous set of parameters: *a* = 3 mm, *b* = 6 mm, (*μ_in_*, *ε_in_*) = (*μ*_0_, 2.2*ε*_0_), (*μ_out_*, *ε_out_*) = (*μ*_0_, 3.8*ε*_0_), (*μ_ext_*, *ε_ext_*) = (*μ*_0_, *ε*_0_), and *m* = 1. In each respective subplot of Figure 6a, Figure 6b and Figure 6c, the real and imaginary parts of the aforementioned eigenvector coefficients: (a) *C*_13_ = $V_{\phi_{TE}}^{ext}$, (b) *C*_14_ = $W_{\phi_{TE}}^{ext}$, and (c) *C*_15_ = $V_{\phi_{TM}}^{ext}$, are plotted versus the effective refractive index *n_eff_* = *β_z_^univ^*/*k*_0_, each of the various values of this latter pertaining to a certain skew angle Φ. The original solved coefficients of (14) are given by circle markers, while the reconstructed ones by polynomial curve-fitting with degree *N* = 6, as of (58), are given by cross markers.

Therefore, for a certain *m* circumferential harmonic and an arbitrarily chosen but thereafter fixed *ϕ*, as well as a certain fixed *z*, all four elements of $\underline{\Pi }$ and thus of $\underline{Z}$ as well [see (55)] are expressible by closed-form analytical functions of *n_eff_*, i.e., we get $Z_{\rho \rho }(n_{eff})$, $Z_{\rho \phi }(n_{eff})$, $Z_{\phi \rho }(n_{eff})$, and $Z_{\phi \phi }(n_{eff})$ in closed-forms, which may then be used in (48). The *n_eff_* in this latter equation, rearranged into the form of

(59) $$\begin{array}{r} F(n_{eff},z)=\left[H_{m}^{\left(2\right)}(-j\alpha_{\rho }b)/{H_{m}^{\left(2\right)}}^{\prime }(-j\alpha_{\rho }b)\right]\eta_{ext}\sqrt{n_{eff}^{2}-1}\\ -\frac{\left\{\begin{array}{l} -\left(Z_{\phi z}Z_{z\phi }-Z_{\phi \phi }Z_{zz}-\eta_{ext}^{2}\right)\\ \pm \sqrt{{\left[\left(Z_{\phi z}Z_{z\phi }-Z_{\phi \phi }Z_{zz}-\eta_{ext}^{2}\right)\right]}^{2}-4Z_{\phi \phi }Z_{zz}\eta_{ext}^{2}} \end{array}\right\}}{2Z_{\phi \phi }}=0 \end{array}$$

expressing a characteristic equation in terms of *n_eff_* and *z*, may then be numerically solved for as roots for one *z* at a time, the solved *n_eff_* pertaining to that certain fixed *z*. Repeating this root solution of *n_eff_* for numerous other *z* locations then yield the string of effective refractive indices to be implemented at various *z*-locations along the rod.

**Figure 7:**
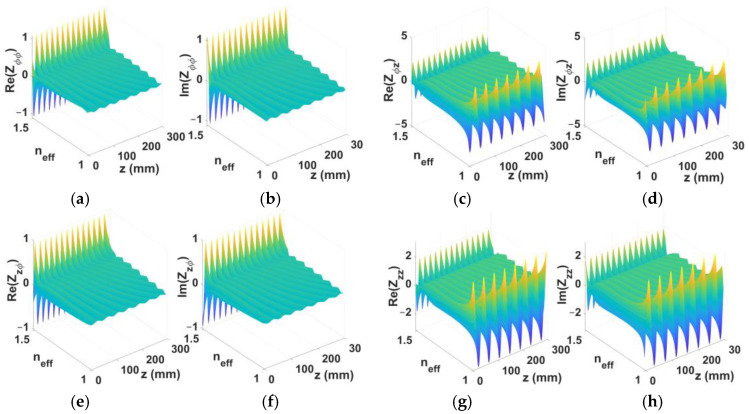
Normalized real and imaginary parts of surface impedance tensor elements: (**a**) Re(*Z_ϕϕ_*), (**b**) Im(*Z_ϕϕ_*), (**c**) Re(*Z_ϕz_*), (**d**) Im(*Z_ϕz_*), (**e**) Re(*Z_zϕ_*), (**f**) Im(*Z_zϕ_*), (**g**) Re(*Z_zz_*), (**h**) Im(*Z_zz_*), contour plotted versus effective refractive index *n_eff_* = *β_z_^univ^*/*k*_0_ and *z*, for single TE beam towards *θ*_0*e*_ = 60°, rod length of 300 mm, with *m* = 1, *f_reson_* = 14 GHz, *a* = 3 mm, *b* = 6 mm, (*μ_in_*, *ε_in_*) = (*μ*_0_, 2.2*ε*_0_), (*μ_out_*, *ε_out_*) = (*μ*_0_, 3.8*ε*_0_), (*μ_ext_*, *ε_ext_*) = (*μ*_0_, *ε*_0_).

As an illustrated example, consider a TE-polarized object plane-wave beam towards *θ*_0*e*_ = 60°, with a rod length of 300 mm, and the same set of *a* = 3 mm, *b* = 6 mm, (*μ_in_*, *ε_in_*) = (*μ*_0_, 2.2*ε*_0_), (*μ_out_*, *ε_out_*) = (*μ*_0_, 3.8*ε*_0_), (*μ_ext_*, *ε_ext_*) = (*μ*_0_, *ε*_0_), *f* = 14 GHz, and *m* = 1. For this case, the normalized real and imaginary parts of the four tensor impedance elements discussed in the previous paragraph, each being a function of both *n_eff_* and *z*, are contour-plotted against these two latter arguments in Figure 7.

**Figure 8:**
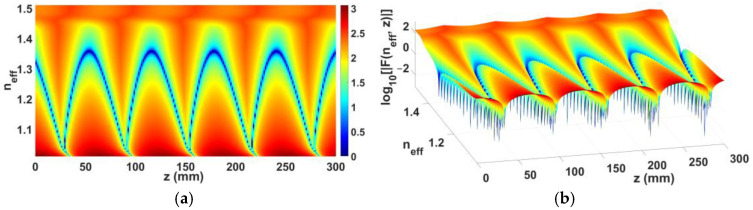
Contour plot of base-10 logarithm, log_10_|*F*(*n_eff_*, *z*)|, of LHS function of characteristic equation in (59), versus *n_eff_* and *z*, for dual beam case of *θ*_0*m*_ = 40° (TM) and *θ*_0*e*_ = 65° (TE), with rod length of 300 mm, with *m* = 1, *f_reson_* = 14 GHz, *a* = 3 mm, *b* = 6 mm, (*μ_in_*, *ε_in_*) = (*μ*_0_, 2.2*ε*_0_), (*μ_out_*, *ε_out_*) = (*μ*_0_, 3.8*ε*_0_), (*μ_ext_*, *ε_ext_*) = (*μ*_0_, *ε*_0_); (**a**) planar top view, and (**b**) perspective view.

For a dual-beam case of which a TM-polarized object plane-wave beam towards *θ*_0*m*_ = 40° and a TE-polarized one towards *θ*_0*e*_ = 65°, and a rod length of likewise 300 mm along with those same latter set of parameters as another showcased example, the contour plot of log_10_|*F*(*n_eff_*, *z*)|, being the base-10 logarithm of the LHS function of the characteristic equation in (59), versus those same two arguments *n_eff_* and *z* as before, is conveyed in two fashions in Figure 8; planar top view in Figure 8a, and perspective view in Figure 8b.

### 5.3. Cylindrical Topology

Under the cylindrical framework with *θ* being the elevation angle from the *z* axis, a single prescribed object beam towards *θ* = *θ*_0#_ with # = 1 is reproduced by a spatial distribution of surface impedances expressed by a function of *z* as

(60) $$jX_{surf}^{1beam}=Z_{surf}^{1beam}=jX\left[1+M\cos\left(k_{0}n_{ave}z-\beta_{z0\#}z\right)\right]$$

where *X* is the real value of the average surface reactance within the range attainable by the parametric span afforded by the considered unit cell of the metasurface, and *M* is the modulation depth, being the division of the difference between the maximum reactance and the average one by the latter (that *X*). Also, *k*_0_ = *k*_0*reson*_ = 2*πf_reson_*√(*μ*_0_*ε*_0_) is the free space wavenumber, *n_ave_* is the average effective refractive index of the span attainable by the parametric range of the considered metasurface unit cell, with any refractive index defined as

(61) $$n_{eff}=\beta_{z,reson}^{univ}/k_{0reson}>1$$

When multiple beams are sought, such as for the example of two beams towards *θ* = *θ*_01_ and *θ*_02_, the surface impedance distribution function is extended to

(62) $$jX_{surf}^{2beams}=Z_{surf}^{2beams}=jX\left\{1+M\cos\left[\left(k_{0}n_{ave}-\beta_{z01}-\beta_{z02}\right)z\right]\right\}$$

With the numerator and denominator of (51) being respectively negative real and positive imaginary for *m* = 1, the cylindrical surface impedance is purely reactive, i.e., *Z_TM_* = *jX_TM_*, leading to

(63) XTMImHm(2)′(−jk0bneff2−1)−Hm(2)(−jk0bneff2−1)η0neff2−1=0

For a certain *X_TM_*, the *n_eff_* can be solved for as roots of this relation. Repeating for numerous reactances and assuming a single root for each, a set of *n_eff_* each corresponding to its own *X_TM_* is yielded, i.e., *n_eff_* as a numerical function of *X_TM_*. By polynomial curve fitting of these data, *n_eff_* as an analytical closed-form polynomial function of *X_TM_* can be obtained, which for a polynomial of degree *N*, assumes the form of

(64) $$n_{eff}(X_{TM})=P_{N}X_{TM}^{N}+P_{N-1}X_{TM}^{N-1}+\cdots +P_{1}X_{TM}+P_{0}$$

where *P_n_* (*n* = *N*, …, 0) are the polynomial coefficients. This (64) conveys the relation between the effective refractive index *n_eff_* and the surface reactance *X_TM_* for any given infinitely-periodic surface, which is a constant *n_eff_* for any given fixed *X_TM_* of a particular periodically textured surface.

Therefore, for modulated surfaces comprising amalgamated periodically-textured surfaces with different properties resulting in varying reactances and thus refractive indices, such as the ones governed by (60) and (62) serving as holographic antennas, the corresponding spatial distributions of the refractive indices associated with those two reactance functions are, according to (64), written as

(65) $$n_{eff}^{\#beams}(z)=P_{N}{\left(X_{surf}^{\#beams}\right)}^{N}+\cdots +P_{1}X_{surf}^{\#beams}+P_{0}$$

expressing the refractive index as a function of the spatial coordinate *z*, with $X_{surf}^{\#beams}$ being either $X_{surf}^{1beam}$ or $X_{surf}^{2beams}$ of (60) or (62), each of these reactances being a function of *z*.

## 6. Computed Results

**Figure 9:**
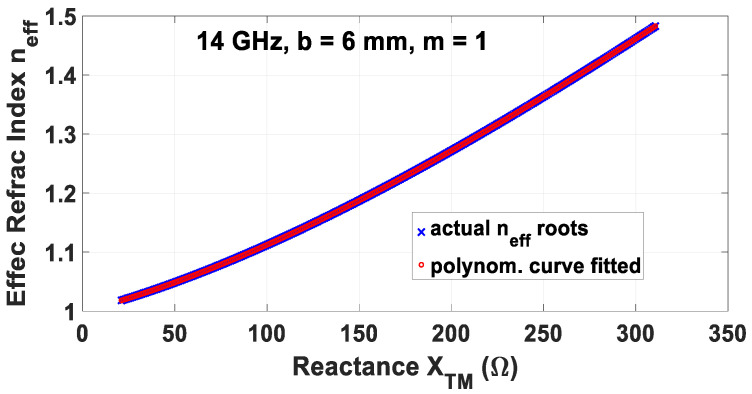
Polynomially curve-fitted graph of *n_eff_* vs. *X_TM_* according to (64).

**Figure 10:**
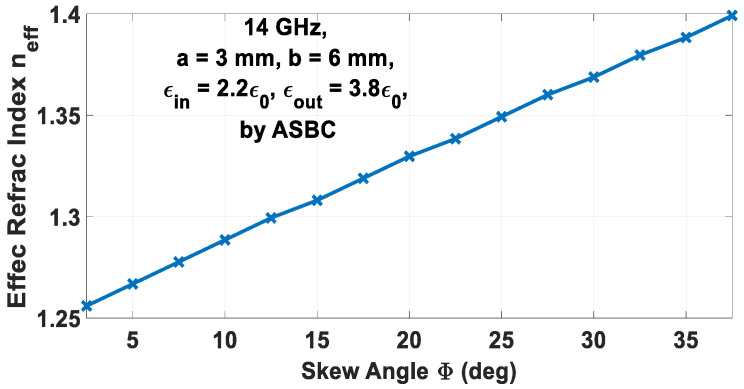
Graph of *n_eff_* vs. skew angle Φ.

Based on (64), the polynomially curve-fitted graph of *n_eff_* versus *X_TM_* is presented in Figure 9 for the case of 14 GHz, *b* = 6 mm, and *m* =1, with all other parameters being the same as those in Section 4. Since any skew angle Φ of the spiral grating at a given frequency corresponds to a particular modal surface wavenumber and thus a certain effective refractive index, the graph of the latter *n_eff_* versus Φ at that same 14 GHz and for those previous parameters is also provided in Figure 10.

### 6.1. Holographic Rod Antennas

In the context of holographic rod antennas, two groups of numerical results are presented. Single-beam designs make up one of them and the other comprises dual-beam cylindrical antennas. For all of them, the operation frequency is 14 GHz and *m* = 1, *a* = 3 mm, *b* = 6 mm, (*μ_in_*, *ε_in_*) = (*μ*_0_, 2.2*ε*_0_), (*μ_out_*, *ε_out_*) = (*μ*_0_, 3.8*ε*_0_), (*μ_ext_*, *ε_ext_*) = (*μ*_0_, *ε*_0_).

#### 6.1.1. Single Beam

(a) *60°, TM polarized*

**Figure 11:**
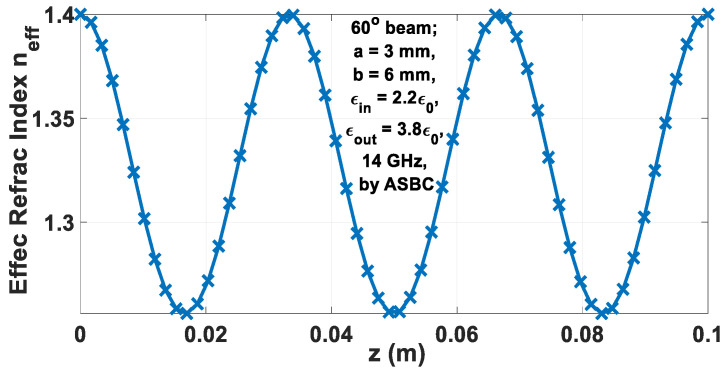
Graph of *n_eff_* vs. *z* according to (65).

**Figure 12:**
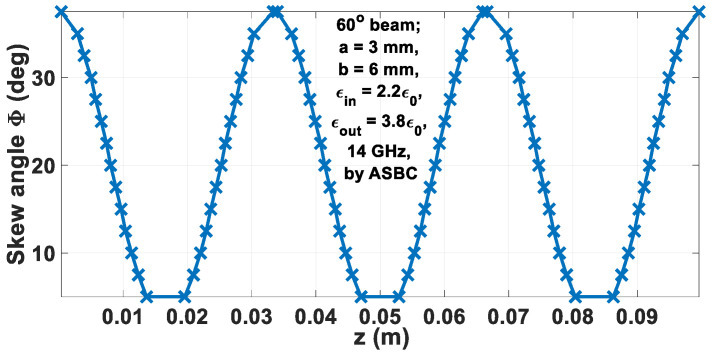
Graph of Φ vs. *z*.

For a 100 mm long rod, the graphs of the refractive index *n_eff_* and the associated skew angle Φ versus the axial length *z* are plotted in Figure 11 and Figure 12, respectively.

**Figure 13:**
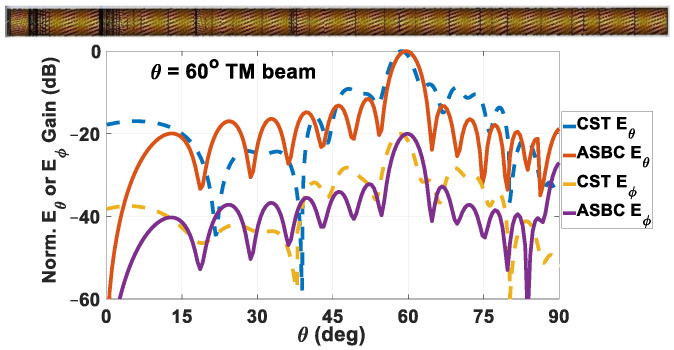
Radiation patterns of holographic rod antenna designed to radiate a single TM-polarized main beam towards *θ*_0*m*_ = 60°, obtained by both solvers, with co-polar *E_θ_* and cross-polar *E_ϕ_* components separately plotted. Schematic of rod antenna shown inset. Maximum directivity = 8.241 dBi, |S_11_| = −14.7324 dB, realized gain = 8.0924 dBi. Radiation efficiency is −3.577 dB.

Generated by the computer program developed according to the ASBC-based analysis along with Fourier aperture integration of Section 3.4, and by CST simulations, the normalized far-field radiation patterns of the holographic rod antenna designed to radiate a single TM polarized beam towards *θ*_0*m*_ = 60° are given in Figure 13, in which the co-polar *E_θ_* and cross-polar *E_ϕ_* components are separately plotted, as annotated in the legend. As observed, the co-polar main beam towards the designated direction is well produced by both solver approaches, as is the cross-polar isolation of about 20 dB. With a maximum directivity of 8.241 dBi and an absolute reflection coefficient |S_11_| of −14.7324 dB, the realized gain is 8.0924 dBi. A radiation efficiency of −3.577 dB is calculated. The schematic of the rod antenna is shown as an inset of the figure.

(b) *40° designed (realized 38°), TE polarized*

**Figure 14:**
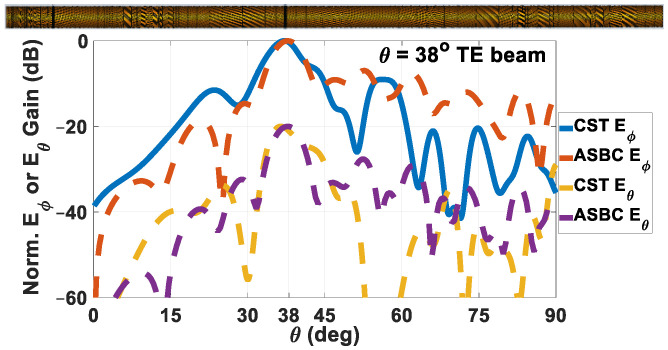
Radiation pattern of holographic rod antenna designed to radiate a single TE-polarized main beam towards *θ*_0*m*_ = 40° (realize 38°), obtained by both solvers, with co-polar *E_ϕ_* and cross-polar *E_θ_* components separately plotted. Schematic of rod antenna shown inset. Maximum directivity = 7.215 dBi, |S_11_| = −15.65 dB, realized gain = 7.0948 dBi. Radiation efficiency is −3.1724 dB.

For the other single-beam case, this time for TE polarization and *θ*_0*e*_ = 40°, the beam patterns obtained by both approaches are presented in Figure 14. The co- and cross-polar components are now respectively *E_ϕ_* and *E_θ_*, which are also separately plotted. Main beams of the co-polar patterns emitted towards *θ* = 38° are portrayed by both solver tools, being a minor squint from the prescribed 40°. The cross-polar level relative to the co-polar radiation is about −19.4 dB. The maximum directivity is 7.215 dBi and the |S_11_| is −15.65 dB, leading to a realized gain of 7.0948 dBi. The computed radiation efficiency is −3.1724 dB. An inset schematic of the rod is also offered in the figure.

#### 6.1.2. Dual Beams

Proceeding to the dual-beam holographic rod antenna, two pairs are reported: (a) TM-polarized beams towards *θ_m_*_01_ = 35° & *θ_m_*_02_ = 50°, and (b) TE-polarized beams towards *θ_e_*_01_ = 40° & *θ_e_*_02_ = 60°.

(a) *35° & 50°, TM polarization*

**Figure 15:**
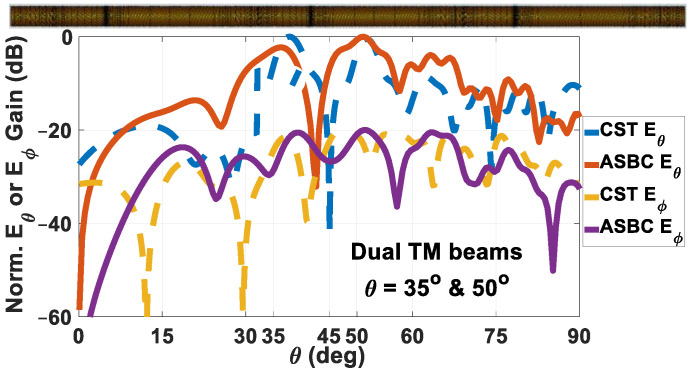
Radiation patterns of holographic rod antenna designed to radiate two TM-polarized beams towards *θ*_0*m*1_ = 35° and *θ*_0*m*2_ = 50°, obtained by both solvers, with co-polar *E_θ_* and cross-polar *E_ϕ_* components separately plotted. Schematic of rod antenna shown above the graph. Maximum directivities towards these two respective beam directions are 7.3 dBi and 6.8 dBi, |S_11_| = −18.1257 dB, respective realized gains = 7.23 dBi and 6.734 dBi. Radiation efficiency is −3.766 dB.

Computed by the analysis-based computer program codes and simulated by CST, the far-field radiation patterns of the dual-beam case of *θ_m_*_01_ = 35° & *θ_m_*_02_ = 50° are presented in Figure 15. As seen, two main lobes towards the prescribed directions are exhibited in the co-polar patterns of both solvers, with the cross-polar radiation being about −20.2 dB compared to the co-polar levels. With directivities of 7.3 dBi and 6.8 dBi respectively for those two beam directions, as well as |S_11_| = −18.1257 dB, the respective realized gains are 7.23 dBi and 6.734 dBi. The radiation efficiency is −3.766 dB.

(b) *40° & 60°, TE polarization*

**Figure 16:**
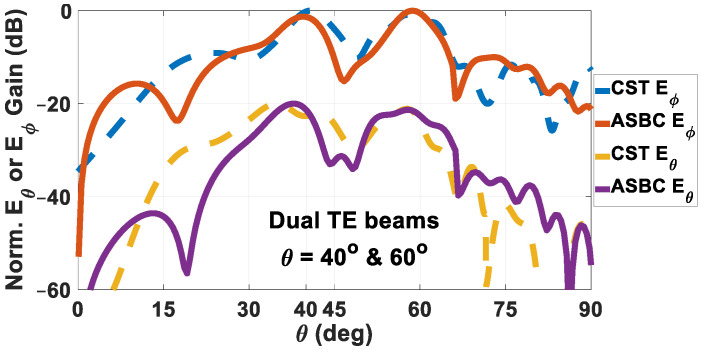
Radiation patterns of holographic rod antenna designed to radiate two TE-polarized beams towards *θ*_0*e*1_ = 40° and *θ*_0*e*2_ = 60°, obtained by both solvers, with co-polar *E_ϕ_* and cross-polar *E_θ_* components separately plotted. Maximum directivities towards these two respective beam directions are 7.1055 dBi and 6.179 dBi, |S_11_| = −15.1757 dB, respective realized gains = 6.9716 dBi and 6.0452 dBi. Radiation efficiency is −2.507 dB.

In similar fashions, the far-field patterns for the TE-polarized dual-beam rod antenna that emits beams towards *θ_e_*_01_ = 40° & *θ_e_*_02_ = 60° are given in Figure 16. Once again, main lobes towards the designated directions are achieved in the co-polar traces of both solvers and the cross-polar decoupling is about 22 dB. The directivities towards these two respective beam directions are 7.1055 dBi and 6.179 dBi, and |S_11_| is −15.1757 dB, resulting in realized gains towards those two directions of 6.9716 dBi and 6.0452 dBi. The radiation efficiency for this case is −2.507 dB. The schematic of the rod antenna for this case is omitted due to space constraint within the graph.

#### 6.1.3. Matching Sections

**Figure 17:**
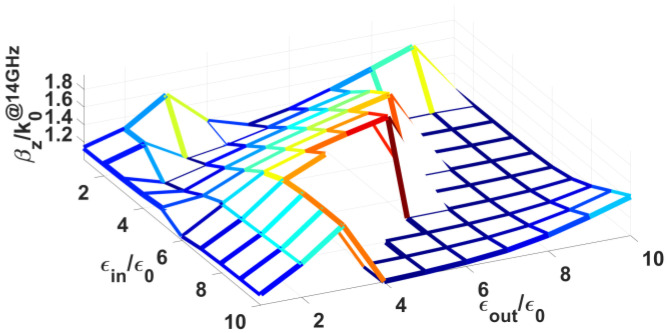
Contour plot of normalized surface wave modal wavenumber *β_z_*/*k*_0_ at 14 GHz of spiral-grated rod with *a* = 3 mm, *b* = 6 mm, Φ = 29°, against relative permittivities (*ε_in_*/*ε*_0_, *ε_out_*/*ε*_0_).

For each of the preceding holographic rod antennas that has been showcased, a coaxial waveguide feed was used in the CST simulations, the outer pipe of which has a radius of 18 mm and its inner rod radius is 6 mm. Placed between this coaxial feed and the first amongst the many grated-rods that make up the antenna is an interconnected rod section, comprising an inner dielectric rod of radius *a* = 3 mm and a permittivity *ε_in_*, sheathed by an outer dielectric pipe of radius *b* = 6 mm, permittivity ε*_out_*, and whose outer surface is wound by the same spiral grating (of the same Φ = 29°) as the first constituent rod of the cylindrical hologram, so as to gradually match this latter to the input coaxial feed. With a dominant TEM modal propagation constant of *β*_0_ = *k*_0_ = 293 rad/m at 14 GHz presented at the input coaxial feed port that is to be matched, the surface wave modal wavenumber *β_z_* of the aforementioned grated rod (obtained by the present ASBC-based modal analysis) at that same frequency and normalized to its *k*_0_, may be contour mesh-plotted against the relative permittivities (*ε_in_*/*ε*_0_, *ε_out_*/*ε*_0_) of its inner rod and outer pipe, as shown by Figure 17. The coordinates at which the normalized wavenumber *β_z_*/*k*_0_ = 1 are found to be (*ε_in_*/*ε*_0_ = 9, *ε_out_*/*ε*_0_ = 4).

With (*ε_in_*/*ε*_0_ = 2.2, *ε_out_*/*ε*_0_ = 3.8) for the entire holographic rod antenna, the dielectric constants of the inner rods of the matching sections are thus to graduate from 2.2 of the first rod section to 9 at the input port of the coaxial feed, while those of the outer pipes vary progressively from 3.8 to 4. As an example of this matching technique, considered here is a simple case of equally-spaced values of (2.2, 3.9, 5.6, 7.3, 9.0) and (3.8, 3.85, 3.9, 3.95, 4.0) for *ε_in_*/*ε*_0_ and *ε_out_*/*ε*_0_, respectively, amounting to just four matching sections, as tabulated in Table 1.

As a further simplification of the design procedure, all four matching sections are assumed to be of the same length, call ℓ*sec*, any one value of which then pertains to a certain matching configuration. Exhibiting just the example case of the TM polarized dual-beam antenna in Section 6.1.2 (a) with (*θ_m_*_01_ = 35°, *θ_m_*_02_ = 50°), various matching topologies are simulated, each characterized by a particular ℓ*sec* and labeled (indexed) by an upper-cased letter, the resultant reflection coefficients S_11_ of which are presented in Table 2. The case without any matching section, associated with ℓ*sec* = 0, is also included.

As seen, the best matching is achieved by the configuration with ℓ*sec* = 2.591305 mm, which lowers the reflection coefficient of the unmatched topology by 1.25 dB.

#### 6.1.4. Summary of Performance

A table that summarizes the performances of the four holographic rod antennas that were showcased in the preceding subsections is offered in Table 3. Aspects that are tabulated include the directivity, |S_11_|, gain, and efficiency.

## 7. Experiments and Measurements

**Figure 18:**
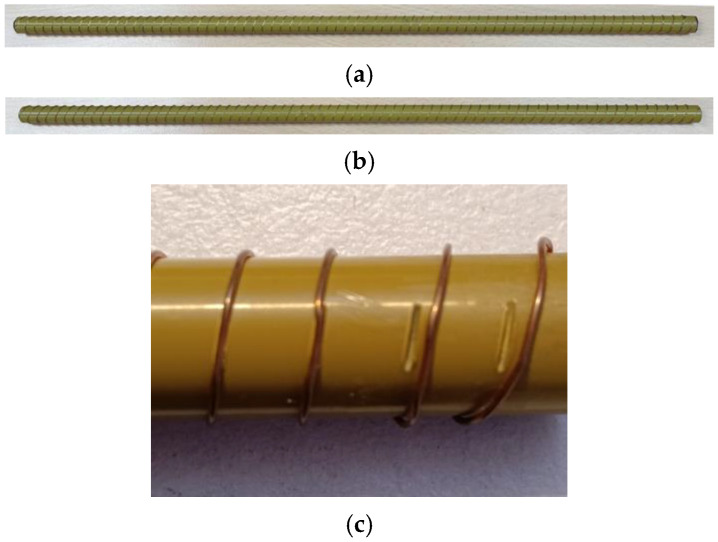
Photographs of the two manufactured prototypes of skewed helical copper wire gratings wound on dielectric pipe sheathed over core dielectric rod (the latter invisible); skew angles (**a**) 20° and (**b**) 30°. Close-up shot in (**c**) of grooves with appropriate tilt angles cut into rod surface for wire to be slotted firmly in place.

Prototypes of two skew-angle cases of copper wire spiral gratings wound on a dielectric pipe wrapped over a core dielectric rod were fabricated, namely Φ = 20° and 30°. For both, a Teflon core rod with a radius of *a* = 3.0 mm and *ε_in_* = 2.1ε_0_ is sheathed by a thermoplastic polyamide-imide (PAI) tube with an outer radius of *b* = 6.0 mm and *ε_out_* = 3.8*ε*_0_. With *μ_in_* = *μ_out_* = *μ_ext_* = *μ*_0_ and a shared common axial period *p* = 5.0 mm, the length of each cylindrical structure is 40 mm and the wire grating has a thickness of 0.3 mm. Photographs of these two manufactured rods, in the above order, are shown in Figure 18a,b.

To accurately wind and bend the spiral grating over the rod such that the correct skew angle is realized poses a mechanical challenge. An innovative maneuver that was undertaken to meet this task is to cut, at appropriate locations, short and shallow grooves into the cylindrical surface of the outer dielectric rod that are tilted according to the skew angles required at those respective places, so that the malleable wire can be slotted into them and be locked into position more firmly, thereby exhibiting and also preserving the correct skew angles. This is photographed in Figure 18c, in which a portion of the grating has been dislodged to reveal two underlying grooves. There are also grooves on the other opposite side of the rod (not visible in the photo) for enhanced stability of the winding.

### 7.1. Modal Dispersion

**Figure 19:**
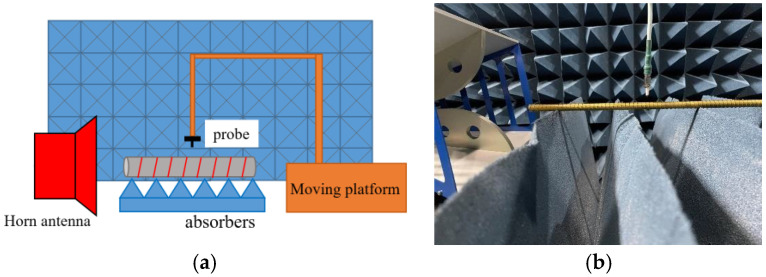
(**a**) Schematic of the measurement setup, and (**b**) photograph of actual experimental scenario for measuring modal dispersion comprising a feed horn antenna and a coaxial probe connected respectively to ports 1 and 2 of a vector network analyzer (not included in the photograph).

For the measurement of modal dispersion, the experimental setup is schematized in Figure 19a, in which a feed horn antenna connected to port 1 of a vector network analyzer emits waves onto the rod, with port 2 connected to a coaxial probe that can be moved along the surface of the spirally grated cylindrical structure. Absorbers are placed around the structure to mitigate interferences from reflected and scattered waves from ambient objects. By sliding the probe along the surface of the rod, the waveform of the field exhibited by the periodically transmitted signal picked up by the probe can be measured, from which the surface wavelength of the oscillation can be determined. The propagation constant along the cylindrical surface is obtained by dividing 2*π* by the latter, quantifying the phase progression per unit distance along the rod. Extracting this measured quantity at various frequencies, a dispersion map can be generated. A photograph of the actual experimental scenario for the measurement of the modal dispersion is provided in Figure 19b.

**Figure 20:**
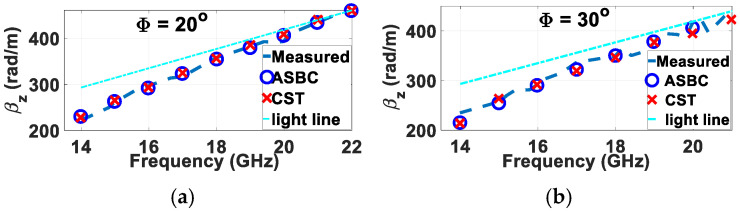
Measured modal dispersion traces of two manufactured helically grated rods of different skew angles compared with theoretical ones predicted by ASBC-based analysis and CST simulations as indicated in legends, both for *a* = 3 mm, *b* = 6 mm, (*μ_in_*, *ε_in_*) = (*μ*_0_, 2.1*ε*_0_ ≈ 2.2*ε*_0_), (*μ_out_*, *ε_out_*) = (*μ*_0_, 3.8*ε*_0_), (*μ_ext_*, *ε_ext_*) = (*μ*_0_, *ε*_0_), for (**a**) Φ = 20°, and (**b**) Φ = 30°.

Presented respectively in Figure 20a,b are the measured modal dispersion curves obtained from experiments for these two rods wound by spiral gratings of skew angles 20° and 30° alongside their corresponding theoretical ones expected of the ASBC-based analysis and CST simulations. As seen, good agreements between theory and experiments are achieved.

### 7.2. Far Field Radiation Patterns

**Figure 21:**
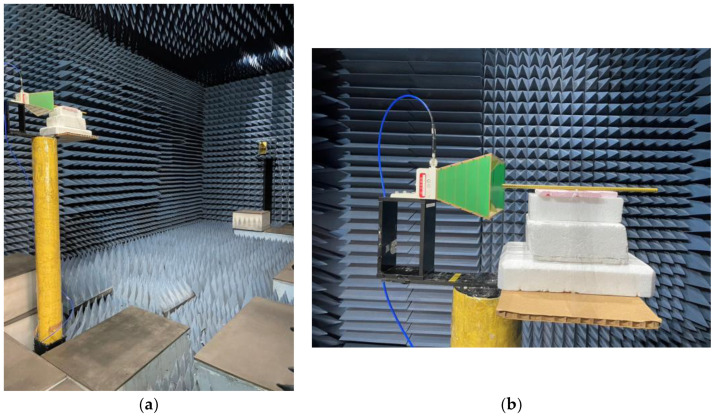
Photographs of experimental setup in an anechoic chamber for measurements of far-field radiation patterns of the manufactured rods; (**a**) overall view of chamber showing feed horn and AUT (grated rod) on rotating platform at near end and receiving horn at far end, and (**b**) closed-up view of grated rod fed by feed horn.

The far-field radiation patterns of the two manufactured rods were measured in an anechoic chamber. Photographs of the experimental scenario and setup are given in Figure 21a,b. Captured at the near end in the former are the feed horn and the AUT (antennas under test) placed on a rotatable platform rack, while visible at the far end is the horn antenna that received the far-field signal. A closed-up view of the feed horn and one of the grated rods is offered in the latter Figure 21b.

**Figure 22:**
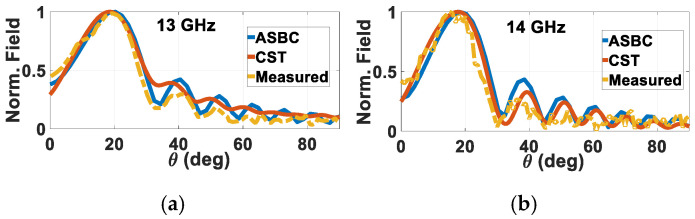
Measured normalized far-field radiation patterns of Φ = 30° rod for *a* = 3 mm, *b* = 6 mm, (*μ_in_*, *ε_in_*) = (*μ*_0_, 2.1*ε*_0_ ≈ 2.2*ε*_0_), (*μ_out_*, *ε_out_*) = (*μ*_0_, 3.8*ε*_0_), (*μ_ext_*, *ε_ext_*) = (*μ*_0_, *ε*_0_), at (**a**) 13 GHz and (**b**) 14 GHz.

**Figure 23:**
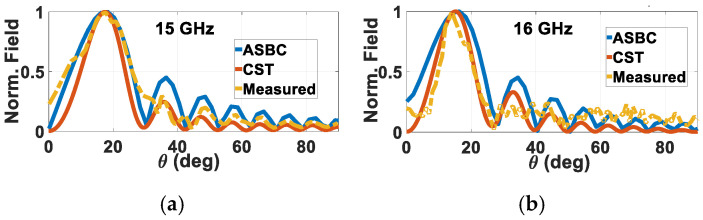
Measured normalized far-field radiation patterns of Φ = 20° rod for *a* = 3 mm, *b* = 6 mm, (*μ_in_*, *ε_in_*) = (*μ*_0_, 2.1*ε*_0_ ≈ 2.2*ε*_0_), (*μ_out_*, *ε_out_*) = (*μ*_0_, 3.8*ε*_0_), (*μ_ext_*, *ε_ext_*) = (*μ*_0_, *ε*_0_), at (**a**) 15 GHz and (**b**) 16 GHz.

The normalized far-field radiation patterns for the Φ = 30° rod measured at 13 and 14 GHz are presented in Figure 22a,b respectively, alongside the respective ones obtained from the analysis and simulations. As seen, the experimental patterns at all considered frequencies match with those of theory. For further validation, measured patterns of the other fabricated helically grated rod with skew angle of Φ = 20° at 15 and 16 GHz are given in Figure 23a,b, again together with their theoretical ones. Good agreement is again observed. As these patterns are of grated rods with uniform skew angles Φ and not yet the holographical ones, their radiative abilities are not expected to be strong, evident by their broader beamwidths and higher sidelobes.

**Figure 24:**
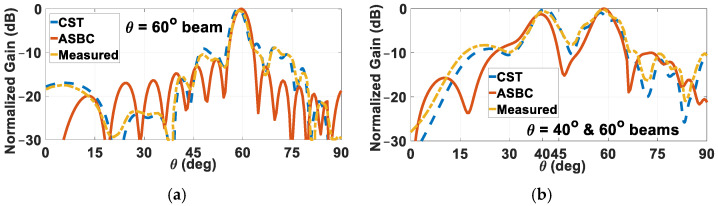
Measured normalized far-field radiation patterns of two holographic rod antennas, designed to radiate (**a**) a single beam towards *θ*_0*m*_ = 60°, and (**b**) double beams towards *θ*_0*e*1_ = 40° and *θ*_0*e*2_ = 60°, both compared with computed ones.

The measured far-field radiation patterns at 14 GHz of two holographic rod antennas, one designated for radiating a single beam towards *θ* = 60°, and the other, a pair of beams towards 40° and 60°, are compared with their computed counterparts in Figure 24a,b, respectively. Good agreements for both cases are observed. Slight deviations of the measured patterns from the theoretical ones are attributed to discrepancies in the fabrication of the rods and measurement errors. Other potential sources of error include the imprecision, with respect to the designated ones in the simulations, of the skew angles of the spiral windings over the manufactured rod prototypes. There could also have been, during the experiments in the anechoic chamber, imperfections with the placements of the grated rod itself as well as the two horns—those of the source and receiver. For any of these three devices, erroneous tilting in terms of pitch, roll, and yaw might have occurred, any of which would cause misalignment and thus inaccurate measurements. Moreover, it is not possible for the 0.3 mm thickness of the helical gratings set in the simulations to be perfectly replicated by that of the wire windings in reality, despite the latter having been measured by a micrometer to be also about 0.3 mm (ranging between 0.297 mm to 0.302 mm, when measured at various locations). Nevertheless, the detrimental effects of this on the accuracy of the measurements are not expected to be severe.

## 8. Conclusions

A dielectric pipe wound by a helical strip grating that is skewed upon stretching two sides of the coil in opposite directions along the axis and sheathed over a core dielectric rod has been analyzed by the asymptotic strips boundary conditions along with the technique of coordinate transformation, all under the analytical framework of vector potentials. The concept of the approach begins with the rolling up of a coordinate-transformed plane in the Cartesian system into that of the cylindrical system, just like rolling up a piece of paper. Results of modal dispersion, field distributions, and radiation patterns computed by the code written based on this method are compared favorably with those simulated by an independent software solver. The latter radiated far-fields are acquired via eigen-modal field solutions provided by the approach that facilitate the Fourier integration of the cylindrical aperture equivalent currents.

The modal field solutions provided by the analysis enable the study and design of holographic cylindrical leaky-wave antennas, which comprise differently grated rod sections that represent the various surface properties required by the cylindrical-type hologram with a modulated reactance surface. Our analysis is distinguished from most other related ones in the literature on holographic cylindrical antennas, which in large proportions, have entailed extensions of initial analyses of conventional planar topologies to their conformal or cylindrical counterparts, thus being somewhat less elegant or robust. Computed results of radiation patterns for such connections of differently skew-grated rods for emitting single and dual beams towards various directions and of different polarizations are compared well with simulations by the software.

Prototypes were also manufactured and measured in terms of modal dispersion and radiation patterns. Results from these experiments agree reasonably with those expected from theory of the analysis and simulations. Slight deviations in the beam directions in the far-field patterns are attributed to discrepancies in the fabrication of the rods and measurement errors.

This work has opened the doors to this type of cylindrical leaky-wave antenna whose surface can be readily retextured by the proposed skew-gratings via mechanical sliding and stretching without the need to change the underlying rod structure, thus offering the possibility of beam reconfiguration and steering. As the helical grating presented herein is also a form of chiral media, our analytical treatment can be extended to the studies of this structure in the research of chiral metamaterials.

## Figures and Tables

**Table 1:** Inner Rod & Outer Pipe Permittivities of Four Matching Sections.

Sec. No.	1	2	3	4
(*ε_in_*/*ε*_0_)	3.9	5.6	7.3	9.0
(*ε_out_*/*ε*_0_)	3.85	3.9	3.95	4.0

**Table 2:** Matching Configurations (14 GHz).

Configuration	ℓ*sec* (mm)	S11 (dB)
No matching	0	−16.874035
A	1.2956525	−17.13643
B	2.591305	−18.1257
C	5.18261	−17.766612
D	10.36522	−17.423341

**Table 3:** Summary of Performance of Four Rod Antenna Cases.

Case	Directivity (dBi)		|S_11_| (dB)	Gain (dBi)		Eff. (dB)
TM 60°	8.241		−14.732	8.0924		−3.577
TE 40°	7.215		−15.65	7.0948		−3.1724
TM 35° & 50°	7.3 (35°)	6.8 (50°)	−18.126	7.23 (35°)	6.734 (50°)	−3.766
TE 40° & 60°	7.1055 (40°)	6.179 (60°)	−15.176	6.9716 (40°)	6.0452 (60°)	−2.507

## Data Availability

The data supporting this study are included within the article.
